# Lipid Nanoparticle Surface Engineering with Heparosan Polysaccharides for Safe and Effective mRNA Delivery *In Vitro* and *In Vivo*

**DOI:** 10.1021/acsami.6c05213

**Published:** 2026-05-12

**Authors:** Yuxin He, Lin Wang, Rameswari Velayutham, Trisha I. Valerio, Samantha D. Ricketts, Yuanhong Sun, Anand C. Annan, James Bowman, Thao Tran, Mobina Mohammadnejad, Kaili Liu, Wei R. Chen, Dixy E. Green, Kar-Ming Fung, Paul L. DeAngelis, Stefan Wilhelm

**Affiliations:** Stephenson School of Biomedical Engineering, University of Oklahoma, Norman, Oklahoma 73019, United States; Stephenson School of Biomedical Engineering, University of Oklahoma, Norman, Oklahoma 73019, United States; Department of Pathology, University of Oklahoma Health Campus, Oklahoma City, Oklahoma 73104, United States; Stephenson School of Biomedical Engineering, University of Oklahoma, Norman, Oklahoma 73019, United States; Department of Pathology, University of Oklahoma Health Campus, Oklahoma City, Oklahoma 73104, United States; Stephenson School of Biomedical Engineering, University of Oklahoma, Norman, Oklahoma 73019, United States; Department of Pathology, University of Oklahoma Health Campus, Oklahoma City, Oklahoma 73104, United States; Stephenson School of Biomedical Engineering, University of Oklahoma, Norman, Oklahoma 73019, United States; Stephenson School of Biomedical Engineering, University of Oklahoma, Norman, Oklahoma 73019, United States; Stephenson School of Biomedical Engineering, University of Oklahoma, Norman, Oklahoma 73019, United States; Stephenson School of Biomedical Engineering, University of Oklahoma, Norman, Oklahoma 73019, United States; Stephenson School of Biomedical Engineering, University of Oklahoma, Norman, Oklahoma 73019, United States; Department of Biochemistry and Physiology, University of Oklahoma Health Campus, Oklahoma City, Oklahoma 73104, United States; Department of Pathology, University of Oklahoma Health Campus, Oklahoma City, Oklahoma 73104, United States; Department of Biochemistry and Physiology, University of Oklahoma Health Campus, Oklahoma City, Oklahoma 73104, United States; Stephenson School of Biomedical Engineering, Institute for Biomedical Engineering, Science, and Technology (IBEST), and Materials Science and Engineering Program, University of Oklahoma, Norman, Oklahoma 73019, United States; Stephenson Cancer Center and Harold Hamm Diabetes Center, University of Oklahoma, Oklahoma City, Oklahoma 73104, United States

**Keywords:** lipid nanoparticles, mRNA, heparosan, drug delivery, nanomedicine, PEG-free formulation

## Abstract

Lipid nanoparticles (LNPs) are clinically established carriers for nucleic acid therapeutics and mRNA-based vaccines. Current LNP formulations often use poly(ethylene glycol) (PEG)-based surface modifications to enhance pharmacokinetics, colloidal stability, and shelf life. However, PEG moieties can elicit anti-PEG immune responses, reactogenicity, and hypersensitivity reactions. Here, we investigated heparosan (HEP), a naturally occurring, biodegradable polysaccharide, for mRNA-LNP surface engineering and mRNA delivery *in vitro* and *in vivo*. We synthesized a library of HEP-coated LNPs and systematically characterized their physicochemical properties, including nanoparticle size, polydispersity, *ζ* potential, and mRNA encapsulation efficiency. We evaluated the delivery performance using two different mRNA payloads in both cultured cells and mouse models. Using HEP-engineered mRNA-LNPs, we demonstrate efficacy comparable to that of PEG-modified counterparts, with minimal tissue damage and negligible immune activation. In summary, our results highlight HEP as an immunologically silent, biocompatible coating agent that enables the formulation of colloidally stable LNPs for safe and effective mRNA delivery *in vitro* and *in vivo*, offering a potential path toward next-generation PEG-free nanomedicines.

## Graphical Abstract

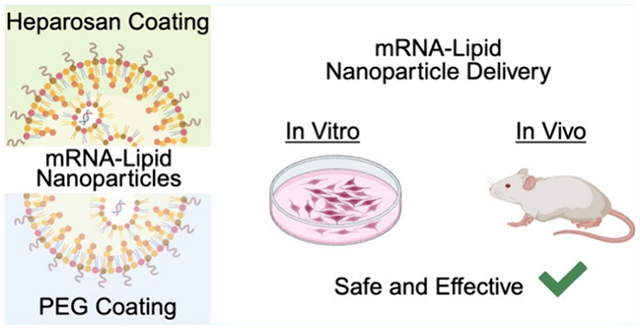

## INTRODUCTION

Lipid nanoparticles (LNPs) have emerged as FDA-approved carriers for nucleic acid–based therapeutics and as platforms for vaccines.^1^ For example, Onpattro (Patisiran) LNPs deliver double-stranded small interfering RNA (siRNA) to the liver for the treatment of polyneuropathy caused by hereditary transthyretin (TTR)-mediated amyloidosis.^2^ These Patisiran siRNA-LNPs accumulate in the liver upon intravenous administration, specifically in hepatocytes, resulting in a decrease in serum TTR levels through RNA interference and a reduction in amyloid deposits in tissues.^2,3^

More recently, mRNA-LNPs have been approved by the FDA for local administration, such as intramuscular injection. These mRNA-LNPs are used as vaccines against COVID-19.^4^ Some mRNA-LNPs have shown promising results in clinical trials for cancer vaccines and clustered regularly interspaced short palindromic repeat (CRISPR)/Cas9-mediated gene-editing applications.^5,6^ Despite this progress, clinically used LNPs formulations face challenges.^7^ One challenge is that some of these formulations use the artificial polymer poly(ethylene glycol) (PEG) for nanoparticle surface modification.^8,9^

The process of coating nanoparticles and drug carriers with PEG polymers is called PEGylation.^10^ The advantages of PEGylation often include enhanced nanoparticle colloidal stability, increased shelf life, and prolonged pharmacokinetics.^11^ However, PEGylated nanoparticles frequently exhibit reduced cellular interactions and cell uptake and have been reported to trigger a phenomenon known as accelerated blood clearance (ABC), particularly when administered repeatedly.^12^ Clinical studies have demonstrated significantly elevated anti-PEG IgG and IgM levels following repeated administration of PEGylated mRNA-LNPs vaccines.^8,13^ Anti-PEG IgG and IgM titers increased 13.1-fold and 68.5-fold, respectively, suggesting an increased risk of hypersensitivity reactions and potentially compromising the safety and efficacy of subsequent doses.^14^

Substituting PEG requires reformulating and revalidating critical quality attributes, including nanoparticle size, polydispersity, encapsulation efficiency, and storage stability. While regulatory agencies curate extensive toxicological and pharmacokinetic data for PEG-based excipients, such as 1,2-distearoyl-*sn*-glycero-3-phosphoethanolamine (DSPE)-PEG 2000 and dimyristoylglycerol (DMG)-PEG2000, comparable data sets for alternative nanoparticle surface modifications remain limited. Various materials, including other synthetic polymers, have been investigated to enhance nanoparticle biocompatibility (Table 1). However, these candidates often exhibit synthetic complexity, electrical charge heterogeneity, or inadequate stability during self-assembly. A fully biodegradable and immunologically silent PEG alternative is needed.^15,16^

To address these limitations, we explored heparosan (HEP), a naturally occurring, biodegradable polysaccharide, as an alternative nanoparticle surface modification agent.^17^ HEP is the unsulfated precursor in the glycosaminoglycan biosynthetic pathway leading to the polysaccharides heparan sulfate on cell surfaces and the anticoagulant heparin.^18^ Composed of repeating *N*-acetylglucosamine-glucuronic acid disaccharides, HEP closely mimics endogenous glycosaminoglycans naturally present on mammalian cell surfaces. This “self-like” structure confers excellent biocompatibility, biodegradability, and negligible immunogenicity.^18^

Nanoparticles coated with HEP form a hydrophilic surface layer that minimizes nonspecific protein adsorption, thereby resulting in PEG-like colloidal stability behavior of the coated nanoparticles. Furthermore, the polysaccharide backbone can be enzymatically degraded by endogenous lyases into nontoxic saccharide building blocks, allowing safe metabolic clearance. Our previous work showed that HEP has no detectable immune response *in vivo*,^17^ and that HEP-coated nanoparticles exhibit reduced protein corona formation and enhanced uptake by antigen-presenting cells compared to PEGylated nanoparticles.^19,20^

In this study, we developed a HEP-based surface engineering strategy for mRNA-loaded LNPs to explore HEP as a potential alternative to PEG. We systematically synthesized and characterized HEP-coated mRNA-LNPs across a broad range of N/P ratios to assess formulation robustness and delivery performance. The effects of HEP surface density on mRNA encapsulation and delivery were evaluated using firefly luciferase mRNA as a model payload. Comparative studies with PEG-modified LNPs were conducted through *in vitro* transfection assays and *in vivo* administration in mice. Additionally, physicochemical characterization and histological analyses were performed to examine formulation stability, biodistribution, and biocompatibility. Finally, we contextualized our findings within the broader landscape of emerging PEG-replacement strategies by comparing LNPs formulation performance, efficacy, toxicity, and practical limitations.

## RESULTS AND DISCUSSION

### Synthesis and Surface Engineering of mRNA-Loaded Lipid Nanoparticles (mRNA-LNPs)

**Figure 1.**
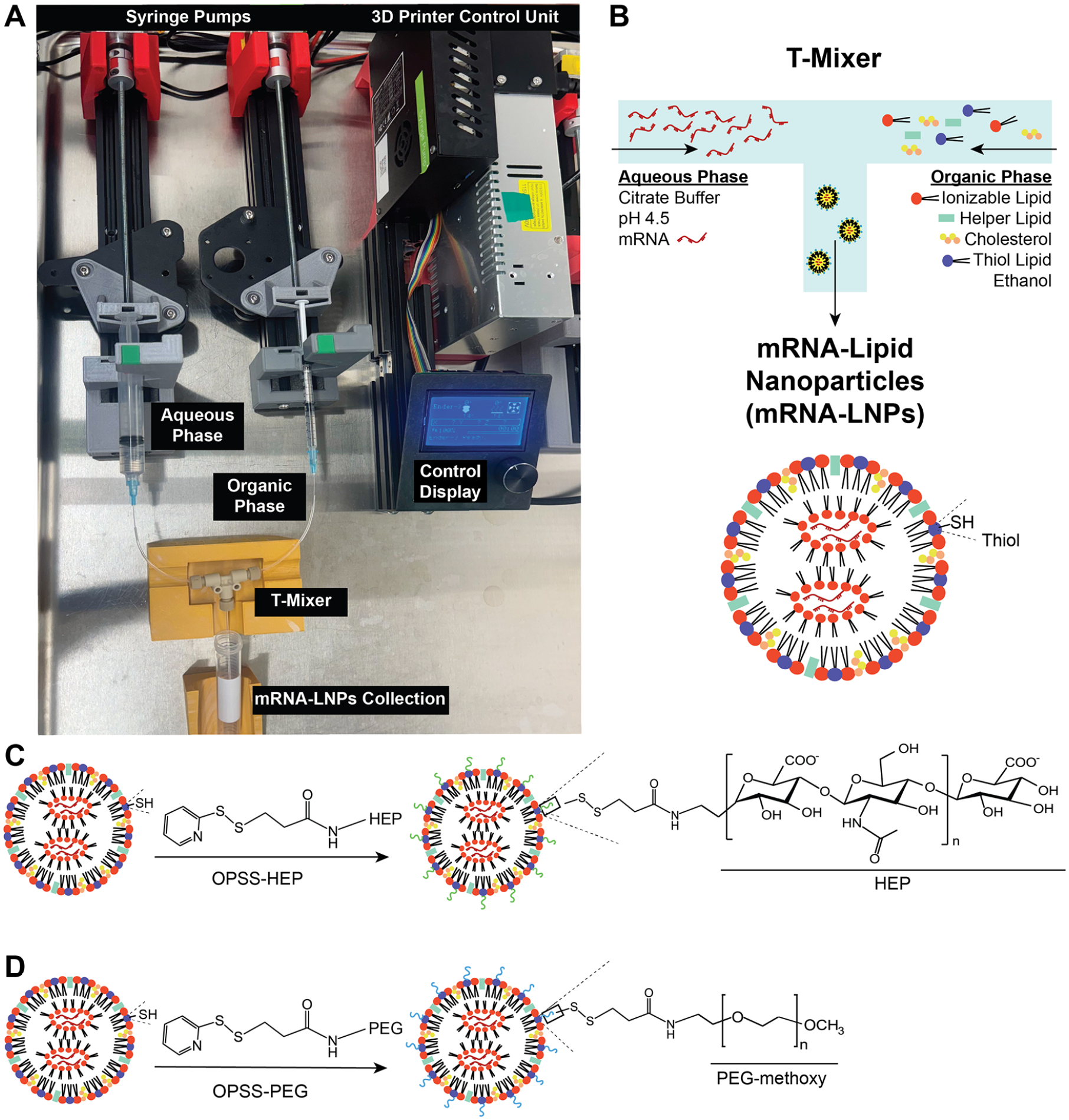
Ender3 3D printer-based fluidic setup for mRNA-LNP synthesis and surface modification. (A) Photograph (top view) of the Ender3 3D printer adapted as a dual-syringe pump system for mRNA-LNP synthesis. Two syringes are loaded with solutions containing lipid components (organic phase) or mRNA (aqueous phase). The 3D-printer control unit drives the syringe pumps. By adjusting the flow rates and flow rate ratios of the organic and aqueous phases, the solutions are combined using a T-mixer, resulting in mRNA-encapsulating LNPs. (B) Simplified schematic of the T-mixer, illustrating how lipid and mRNA streams merge and mix to generate mRNA-loaded LNP formulations. Note: A thiol-terminated lipid was added to the lipid mixture for downstream LNP surface modification. (C and D) Schematic representation of LNP surface modification with HEP or PEG polymers. Upon pH adjustment of the mRNA-LNPs suspension, OPSS-HEP or OPSS-PEG was added to form the surface modification.

We prepared mRNA-LNPs using a home-built 3D-printer-based fluidic mixing system (Figure 1A).^21^ We selected the following lipid formulation, which consisted of (6*Z*,9*Z*,28*Z*,31*Z*)-heptatriaconta-6,9,28,31-tetraen-19-yl 4-(dimethylamino)butanoate (DLIN-MC3-DMA) ionizable lipid, 1,2-distearoyl-*sn*-glycero-3-phosphocholine (DSPC) helper lipid, cholesterol, and 16:0 phosphatidylthioethanol (thiol-lipid), combined at a defined molar ratio (DLIN-MC3-DMA:DSPC:cholesterol:thiol-lipid = 50:10:38.5:1.5) and optimized for efficient nucleic acid encapsulation and delivery.^22^

As illustrated in Figure 1B, the fluidic system uses a T-mixer setup to combine two liquid streams: (i) the aqueous phase consisted of mRNA dissolved in citrate buffer (10 mM, pH ~ 4.5); (ii) the organic phase consisted of the lipid mixture dissolved in ethanol under controlled flow rates. Upon mixing, the mRNA and lipid components self-assemble into mRNA-LNPs at varying N/P ratios. We established different N/P ratios by maintaining a constant number of ionizable lipid amine groups (N) while varying the amount of mRNA (P). The optimal N/P ratio for mRNA encapsulation and delivery is highly formulation-dependent and varies with ionizable lipid structure, surface chemistry, and post-formulation modification strategies. To evaluate the robustness of HEP surface engineering across formulation conditions, we examined a range of N/P ratios (5, 10, and 100), spanning commonly used values and extended conditions reported for ionizable lipid-based LNPs. While N/P ratios around 3–8 are often reported for siRNA and mRNA delivery, higher N/P ratios have been widely employed in ionizable lipid LNP systems to improve encapsulation, stability, and *in vivo* performance (e.g., N/P 10–20 and higher).^23,24^

In this study, we incorporated a thiol-lipid into the LNP formulation to introduce chemically reactive thiol groups for downstream nanoparticle surface engineering.^25,26^ These thiol moieties enable the covalent attachment of orthopyridyl disulfide (OPSS)-functionalized polymers through disulfide-mediated surface attachment.^20^ As illustrated schematically in Figure 1C,D, we functionalized the surface of the thiol-lipid modified mRNA-LNPs with either 13-kDa OPSS-terminated HEP (OPSS-HEP) or 10-kDa OPSS-terminated PEG (OPSS-PEG) polymers. We selected HEP based on our previous findings demonstrating its excellent ability to provide nanoparticle colloidal stability, biocompatibility, lack of immunogenicity, and enhanced cellular interactions with antigen-presenting cells.^19,20,27^ In contrast to ligand-conjugated LNPs, which typically require multistep synthesis, covalent coupling chemistry, purification, and batch-specific optimization, the HEP coating described here is introduced through a single post-formulation adsorption step. This approach avoids modification of the core LNP composition and preserves the underlying formulation workflow. The 13-kDa OPSS-HEP polymers were synthesized in-house following established protocols, while commercially available 10-kDa OPSS-PEG was used as a control to ensure comparable molecular weight and conjugation chemistry.^17,18,20^ We selected PEG as the benchmark polymer due to its widespread clinical use in nanomedicine.^14,28^

The HEP surface functionalization in this study was achieved via disulfide-based bioconjugation, which introduces both advantages and potential limitations. Disulfide linkages are generally stable under extracellular oxidative conditions but are susceptible to cleavage in reductive environments, such as those containing elevated glutathione levels in, including some intracellular compartments.^29^ As a result, premature cleavage of the disulfide bond following *in vivo* administration cannot be fully excluded and may lead to partial loss of surface modification prior to the cellular uptake of the nanoparticles. However, this dynamic behavior may also be beneficial, as cleavage in endosomal or cytosolic environments could expose the underlying LNP components, potentially enhancing intracellular delivery.^30,31^

Compared to nanoparticle modification strategies in which functionalized lipids are incorporated during nanoparticle formulation, the post-conjugation approach used in our study offers great design flexibility and enables surface modification of preformed LNPs without altering core formulation parameters.^32^ This modular strategy can be advantageous for rapid screening of different surface chemistries and potential surface ligand candidates. Further optimization of linker chemistry or comparison with prefunctionalized lipid incorporation may be warranted to balance stability and functional performance in future studies.^33^

### Characterization and Design Optimization of mRNA-LNPs (N/P 100)

We formulated mRNA-LNPs using a fixed lipid-to-mRNA ratio of N/P 100. This ratio is often used for LNP formulations to ensure complete mRNA encapsulation and efficient intracellular delivery.^34,35^ Because mRNA carries a dense negative charge, an excess of ionizable lipid is required to promote stable nanoparticle formation.^36^ While low N/P ratios (less lipid per mRNA) may lead to decreased mRNA encapsulation and colloidal instability, high N/P ratios provide excess ionizable lipids, enabling tighter packing, smaller nanoparticle size, and higher encapsulation efficiency. A potential downside of high N/P ratios is that they may increase cytotoxicity and alter the biodistribution of LNPs.^32^ We quantified mRNA encapsulation using a RiboGreen fluorescence-based assay (Figure S1).

Typically, N/P values near 100 yield compact, monodisperse nanoparticles [60–100 nm; polydispersity index (PDI) < 0.2] with reproducible colloidal stability.^32^ The ionizable lipid can further facilitate endosomal escape through a recently discovered vesicle budding-and-collapse (VBC) mechanism.^37^ Importantly, ionizable lipids such as DLIN-MC3-DMA remain largely neutral at physiological pH, reducing cytotoxicity while maintaining transfection efficiency at this ratio.^32^

**Figure 2.**
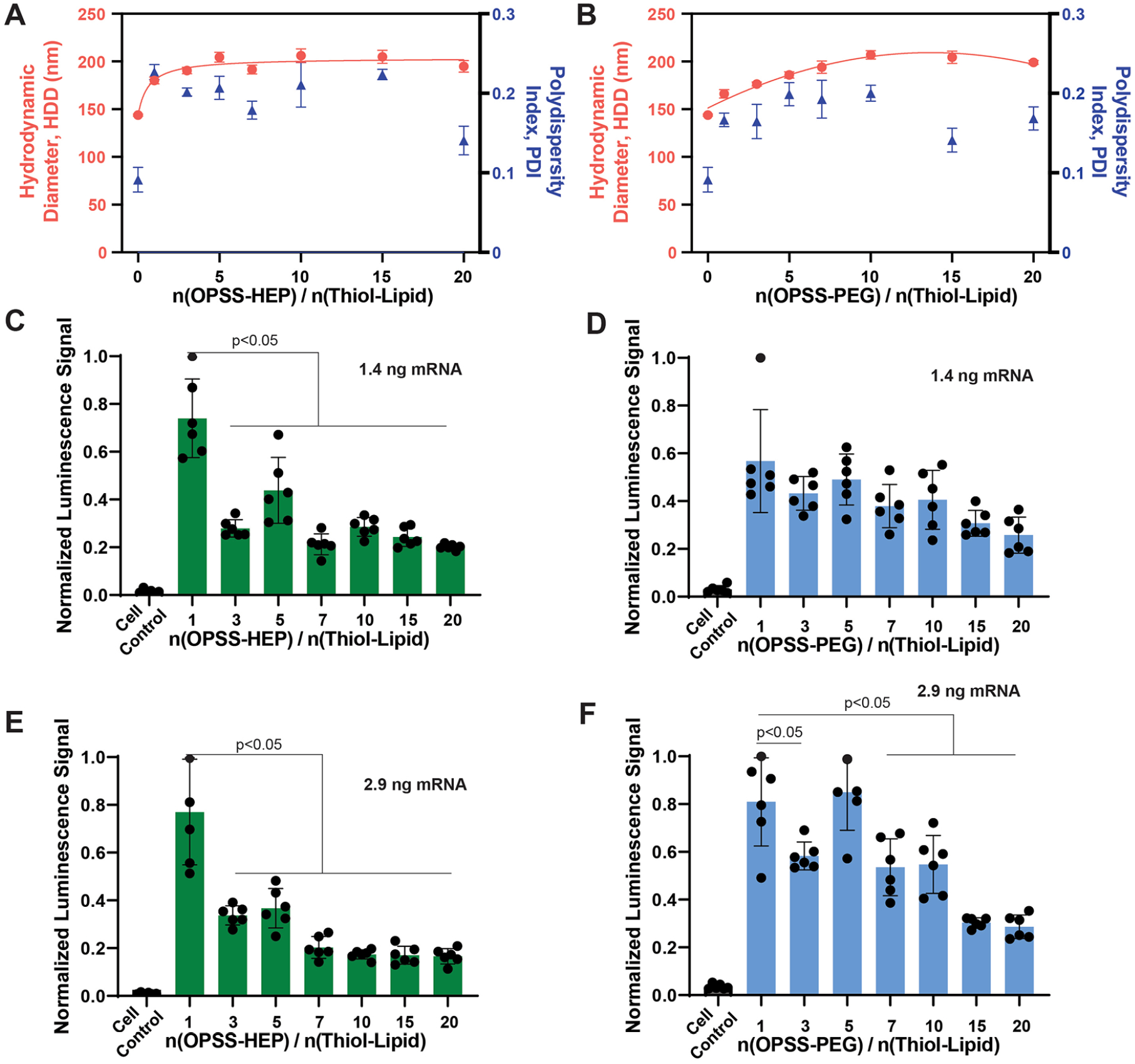
Characterization and LNP design optimization using an *in vitro* luciferase mRNA-based bioluminescence readout. (A) HDD (red symbols) and PDI (blue symbols) of mRNA-LNPs with varying HEP polymer-to-thiol lipid molar ratios, measured by DLS after dialysis. Data represent mean ± standard deviation (*n* = 3). The red solid line is a guide to the eye. (B)HDD(red symbols) and PDI (blue symbols) of mRNA-LNPs with varying PEG polymer-to-thiol lipid molar ratios, measured by DLS after dialysis. Data represent mean ± standard deviation (*n* = 3). The red solid line is a trendline. RAW 264.7 murine macrophages incubated with 1.4 ng of luciferase mRNA delivered by LNPs coated with different HEP-to-thiol-lipid molar ratios (C) or different PEG-to-thiol-lipid molar ratios (D). RAW 264.7 macrophages incubated with 2.9 ng of luciferase mRNA delivered by LNPs coated with different HEP-to-thiol-lipid molar ratios (E) or different PEG-to-thiol-lipid molar ratios. (C–F) TheRAW264.7 macrophages were incubated for 24 h. The bars represent mean ± standard deviation (*n* = 6). Statistical analysis was performed using one-way ANOVA with Tukey’s HSD posthoc test.

On the basis of these design principles, we formulated LNPs at N/P 100 and subsequently introduced surface modifications using HEP or PEG conjugation. Firefly luciferase mRNA served as a representative payload. The unmodified LNPs exhibited an average hydrodynamic diameter (HDD) of ~145 nm with a PDI of 0.09 (Figure 2A,B). We then systematically varied the OPSS-HEP-to-thiol-lipid or OPSS-PEG-to-thiol-lipid molar ratio from 1:1 to 20:1 and assessed changes in HDD and PDI by dynamic light scattering (DLS). Both HEP- and PEG-modified LNPs exhibited increased HDD compared to unmodified controls, confirming successful surface conjugation. Beyond a 10:1 ratio, the nanoparticle size plateaued at ~200 nm, while PDI values remained within <0.2, indicating consistent particle size distribution following surface modification and dialysis. These physicochemical characteristics indicate that colloidal stability is maintained under the tested conditions.

To further assess how the OPSS-HEP (or OPSS-PEG)-to-thiol-lipid molar ratio affected the functional delivery of mRNA, we next incubated RAW264.7 murine macrophages *in vitro* for 24 h with mRNA-LNPs modified at various surface ratios, delivering either 1.4 ng (14 ng/mL) or 2.9 ng (29 ng/mL) of firefly luciferase mRNA per condition (Figure 2C–F). Using an established luciferase bioluminescence assay,^21^ we observed that mRNA-LNPs modified with 1:1 OPSS-HEP (or OPSS-PEG)-to-thiol-lipid molar ratio produced a robust bioluminescence signal, indicating efficient functional mRNA delivery and translation (Figure 2C–F). Notably, HEP-mRNA-LNPs showed greater transfection efficiency than PEG-mRNA-LNPs at this ratio, highlighting the enhanced transfection capability of HEP-coated LNPs. At higher surface modification ratios, excessive HEP or PEG coating may impede cellular uptake and endosomal escape by sterically shielding the LNP surface and limiting membrane interactions. This effect may reduce functional mRNA delivery despite improved colloidal stability, thereby diminishing transfection efficiency, potentially due to decreased VBC efficiency.^37^

On the basis of our initial screening results, we selected the 1:1 OPSS-HEP (or OPSS-PEG)-to-thiol-lipid molar ratio for subsequent *in vitro* and *in vivo* studies. Maintaining this consistent ratio allowed us to directly compare the HEP- and PEG-modified LNPs. We also selected different N/P ratios throughout this study to demonstrate the flexibility and modularity of our LNPs synthesis approach. We initially used an N/P ratio of 100 for formulation screening to ensure robust mRNA complexation.

For subsequent *in vitro* studies, we selected an N/P ratio of 10 to better reflect commonly used conditions in the literature. For *in vivo* experiments, we selected an N/P ratio of 5 to reduce the total lipid amount and improve tolerability, as lower N/P ratios are generally preferred for *in vivo* administration.^38^

### *In Vitro* Cellular Toxicity and Efficacy Assessment of HEP-and PEG-Modified Firefly Luciferase mRNA-LNPs (N/P Ratio of 100)

**Figure 3.**
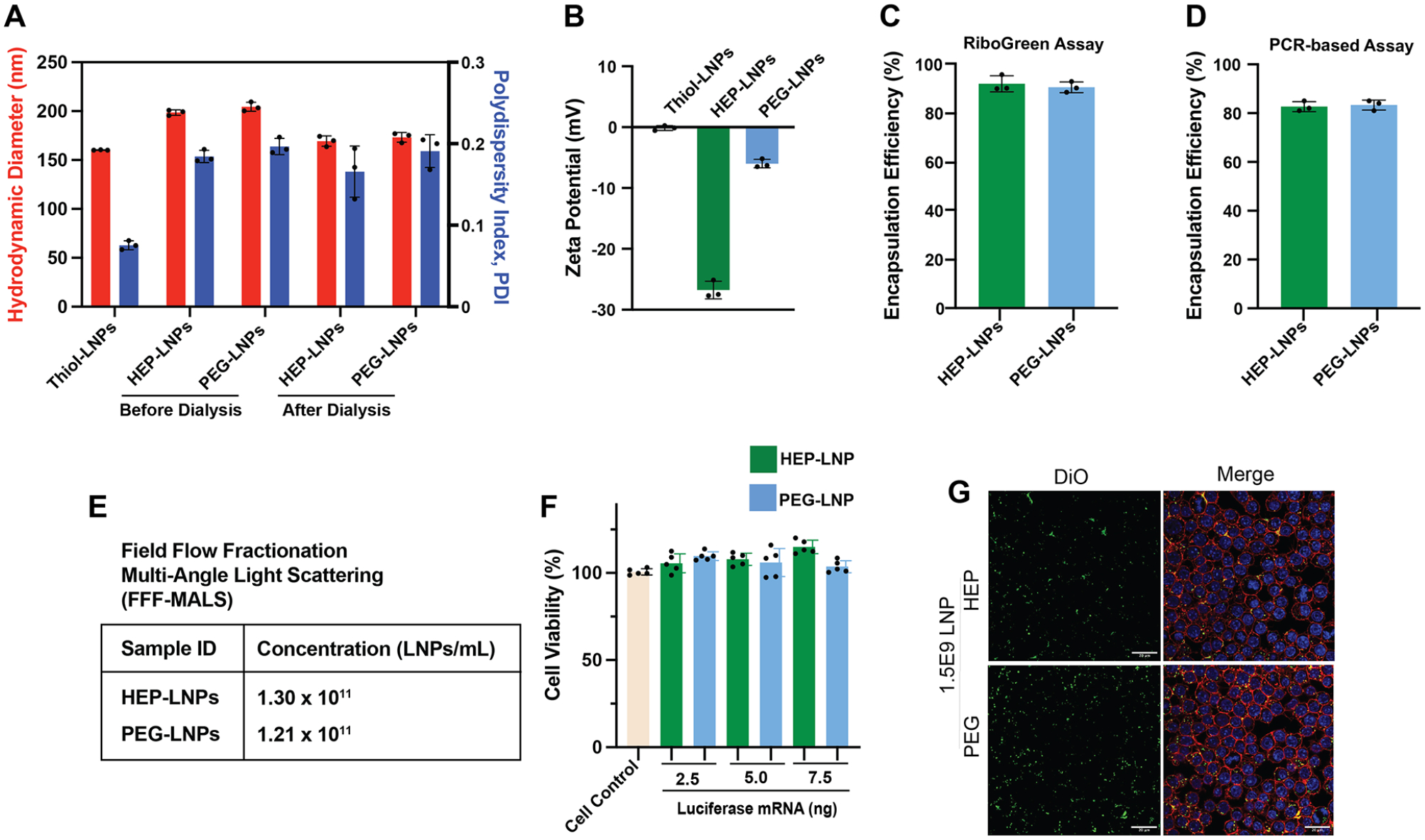
Characterization and cellular toxicity assessment of HEP- and PEG-modified firefly luciferase mRNA-LNPs with an N/P ratio of 100. (A) HDD and PDI of mRNA-LNPs measured by DLS. Data are shown as mean ± standard deviation (*n* = 3). (B) *ζ* potential of HEP- and PEG-coated LNPs measured via electrophoretic mobility (measured in 10 mM HEPES buffer, pH 7.4). Data are shown as mean ± standard deviation (*n* = 3). (C) RiboGreen assay results for mRNA encapsulation efficiency (%EE) of mRNA-LNPs. Data are shown as mean ± standard deviation (*n* = 3). (D) PCRbased assay results for mRNA %EE of mRNA-LNPs. Note: The data represent intact mRNA encapsulated within LNPs. Data are shown as mean ± standard deviation (*n* = 3). (E) Concentration of mRNA-LNPs quantified by FFF-MALS. (F) XTT-based assay to assess the cell viability of RAW 264.7 murine macrophage cells treated with mRNA-LNPs at doses of 2.5, 5.0, and 7.5 ng mRNA, after 24 h of incubation. Data represent mean ± standard deviation (*n* = 5). One-way ANOVA analysis showed no significant differences (*p* > 0.05) in the cell viability between treatment groups. (G) CLSM images of RAW 264.7 murine macrophages incubated for 24 h at 37 °C with HEP- or PEG-coated luciferase mRNA-LNPs (1.5 × 109 particles per well). LNP particle numbers were quantified by FFF-MALS. The mRNA-LNPs were labeled with the lipophilic fluorescent dye Vybrant DiO (green). After incubation, cells were washed, fixed, and stained for imaging. Cell nuclei were counterstained with 4′,6-diamidino-2-phenylindole (DAPI, blue), and cell membranes were labeled with wheat germ agglutinin conjugated to CF633 (WGA-CF633, red). The merged images show DiO-labeled LNPs distributed within the cytoplasmic region. Scale bars = 20 *μ*m. (Same images as Figure S2, 1.5 × 109 LNPs group, green and merged channels.)

Next, we performed a comprehensive physicochemical analysis of mRNA-LNPs modified with 1:1 OPSS-HEP (or OPSS-PEG)-to-thiol-lipid molar ratio (Figure 3). We observed that the HDD increased by 38 or 44 nm upon conjugation of OPSS-HEP or OPSS-PEG, respectively (Figure 3A). Additionally, compared to the initial thiol-modified LNPs, the *ζ* potential of HEP-modified LNPs decreased by ~27 mV, while the *ζ* potential of PEG-modified LNPs remained near neutral as expected (~5 mV; Figure 3B).^19,20^ These results indicate the successful LNPs surface modification with OPSS-HEP or OPSS-PEG moieties.

To confirm successful surface conjugation of HEP onto LNPs, polyacrylamide gel electrophoresis (PAGE) was performed in three independent experiments to quantify surface-associated HEP and determine HEP density per particle (Figure S2). Across all replicates, a consistent value of 0.12 *μ*g HEP/*μ*L of LNP suspension was obtained. Based on these measurements and LNP nanoparticle concentration determined by field-flow fractionation coupled with multiangle light scattering (FFF-MALS), this corresponds to approximately 56 HEP molecules per LNP, equivalent to ~1 HEP molecule per 1400 nm^2^ of LNP nanoparticle surface area. It should be noted that centrifugation was required during sample preparation. Given the structural sensitivity of thiol-lipid-containing LNPs, partial particle disruption may have occurred during this process, potentially leading to underestimation of surface-associated HEP. Nevertheless, HEP was consistently detected on the LNP surface across all independent experiments, supporting successful surface coating.

We further determined the mRNA encapsulation efficiency using a RiboGreen fluorescence-based assay (Figure S1), yielding ~90% EE for HEP- and PEG-modified mRNA-LNPs (Figure 3C). The EE was determined using a RiboGreen-based fluorescence assay, which quantifies total RNA but does not assess mRNA integrity. To address this limitation, we employed a polymerase chain reaction (PCR)-based assay using flanking primers to evaluate the fraction of intact mRNA. This analysis indicated that approximately 84% of the encapsulated mRNA remained intact (Figure 3D).^39^

Next, we determined the LNPs’ number concentration using FFF-MALS and observed that both groups (HEP-LNPs and PEG-LNPs) exhibited similar LNPs concentrations (~1 × 10^11^ LNPs/mL; Figure 3E). Cell viability testing using RAW264.7 murine macrophages showed consistent results (~100% viability) for both HEP- and PEG-modified mRNA-LNPs groups, indicating no toxicity up to 7.5 ng (75 ng/mL) of mRNA over 24 h of exposure (Figure 3F).

Collectively, these data suggest that the synthesized LNPs were of high quality with excellent safety profiles. We further confirmed that both HEP- and PEG-modified mRNA-LNPs were effectively internalized by RAW264.7 macrophages in a concentration-dependent manner. Equal particle numbers (1.0 × 10^9^ or 1.5 × 10^9^ DiO-labeled LNPs per well) were applied, and cellular uptake was visualized by confocal laser scanning microscopy (CLSM), as shown in Figures 3G and S3. Both formulations exhibited comparable intracellular fluorescence intensities, indicating similar levels of cellular uptake. Given this comparable uptake efficiency, the enhanced luciferase transfections observed for HEP-coated LNPs in Figure 2 may arise from improved intracellular mRNA release and translation efficiency. Further studies are needed to validate this mechanism and its relationship to the recently proposed VBC mechanism.^37^

We further validated the reproducibility and reliability of our LNP formulations (Figure S4). We synthesized firefly luciferase mRNA-LNPs with an N/P ratio of 100 and 1:1 OPSS-HEP- or OPSS-PEG-to-thiol-lipid ratio. We then quantified the number concentrations of LNPs using FFF-MALS (Table S1). Across all three independent replicates, the LNPs’ number concentrations were consistent (~1 × 10^11^ LNPs/mL), and the HDD and PDI matched closely between the replicates (Figure S4A–C). We quantified mRNA %EE and observed consistent ~90%EE across all formulations (Figure S4D–F), indicating robust, reproducible mRNA loading.

To assess the functional mRNA delivery, we conducted firefly luciferase bioluminescence assays. Three biological replicates of RAW264.7 macrophages were treated for 24 h with both HEP-and PEG-modified mRNA-LNPs delivering increasing doses of firefly luciferase mRNA up to 7.5 ng (75 ng/mL). Across the three biological replicates, comparable luminescence signals were obtained. Following the increase in mRNA dose, we observed a corresponding increase in bioluminescence signal intensity, with HEP-mRNA-LNPs consistently showing trans fection efficiencies comparable to or higher than those of their PEG-mRNA-LNPs counterparts (Figure S4G–I).

### Synthesis and Characterization of Surface-Modified mRNA-LNPs (N/P 10)

To further demonstrate the versatility of our mRNA-LNP synthesis approach, we formulated mRNA-LNPs at a reduced N/P ratio of 10 to evaluate formulation behavior and delivery performance. Although high N/P ratios (e.g., N/P 100) are often used in experimental settings to maximize delivery efficiency, ratios between 5 and 10 are more commonly used in translational and clinical research due to improved safety and reduced lipid-associated toxicity.^34^ Lowering the N/P ratio decreases the excess ionizable lipid content, thereby minimizing potential cytotoxicity and improving biocompatibility while maintaining sufficient electrostatic interaction for efficient mRNA encapsulation.

**Figure 4.**
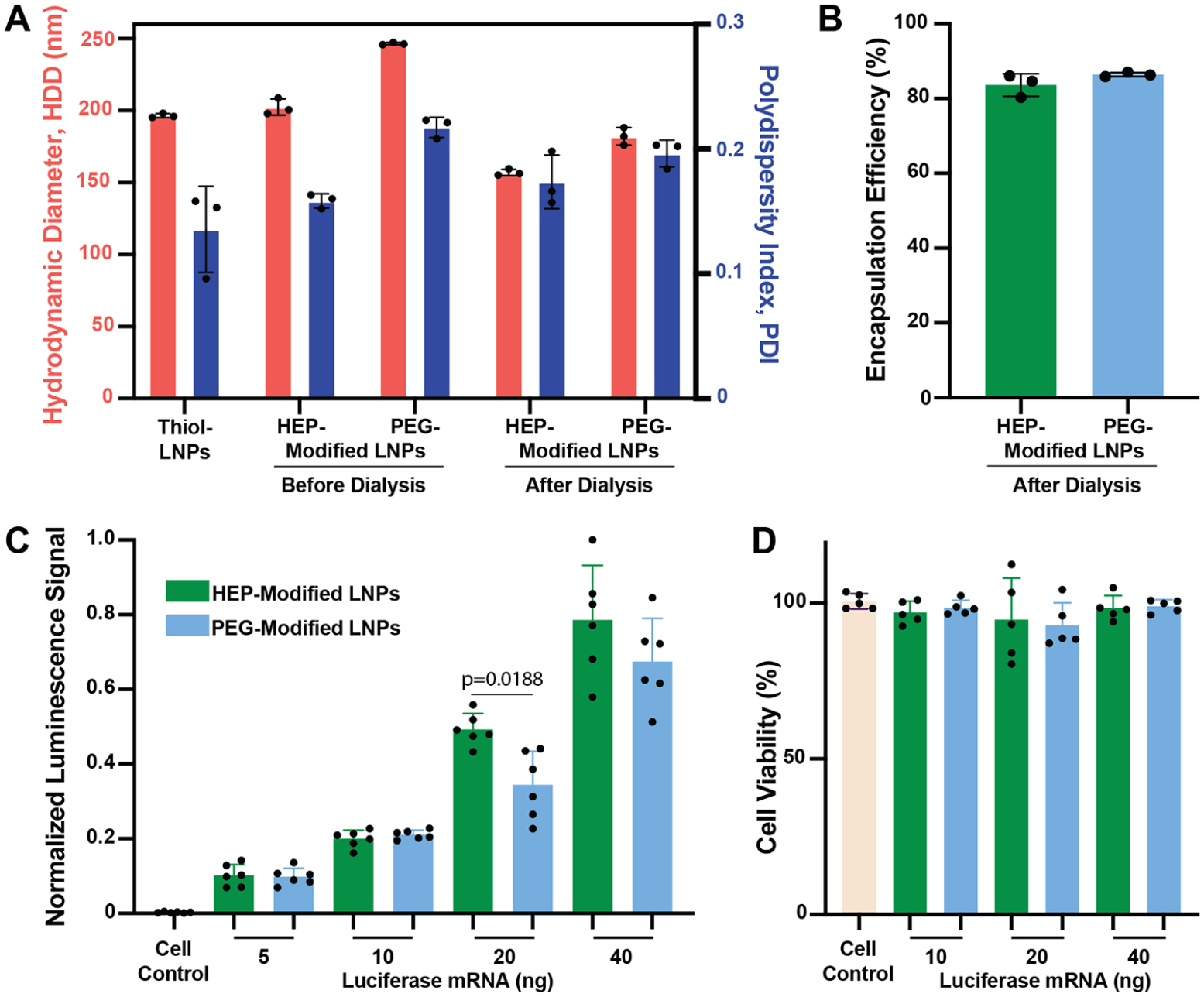
Characterization of HEP- and PEG-modified luciferase mRNA-LNPs with a N/P ratio of 10 for *in vitro* transfection. (A) HDD and PDI of mRNA-LNP formulations, measured by DLS. The bars represent mean ± standard deviation (*n* = 3). (B) %EE of the corresponding mRNA-LNPs. The bars represent mean ± standard deviation (*n* = 3). (C) Transfection of RAW264.7 murine macrophages with the corresponding mRNA-LNPs after 24 h of incubation. The bars represent mean ± standard deviation (*n* = 6). A statistical difference was observed for the 20-ng condition (*p* = 0.0188) using a *t* test. (D) Cell viability (XTT) assay of RAW264.7 murine macrophages treated with the indicated mRNA-LNP groups and incubated for 24 h. The bars represent mean ± standard deviation (*n* = 5). A one-way ANOVA was used to compare cell viability. No statistically significant differences (*p* > 0.05) were observed among the experimental samples.

Using DLS analysis, we observed that the resulting mRNA-LNPs (N/P 10) HDD and PDI closely matched those observed for mRNA-LNPs with N/P 100 (Figure 4A). The mRNA encapsulation efficiencies remained high, exceeding 80% (Figure 4B). Our FFF-MALS analysis revealed a slightly decreased LNPs number concentration with ~6 × 10^10^ LNPs/mL for both HEP- and PEG-modified LNPs (Table S2). To evaluate the transfection efficiency, RAW264.7 murine macrophages were incubated for 24 h with increasing mRNA doses up to 40 ng (400 ng/mL) delivered via either HEP- or PEG-modified mRNA-LNPs. We observed that the detected bioluminescence signal intensities increased in a dose-dependent manner with increasing mRNA dose. As anticipated, increasing the mRNA concentration resulted in proportionally stronger bioluminescence signals. Additionally, the HEP-modified mRNA-LNPs tended to yield higher signal intensity at higher mRNA concentration than their PEG-modified mRNA-LNPs counterparts (Figure 4C), indicating improved mRNA delivery efficiency. We also performed XTT-based cell viability assays, which confirmed that none of the tested LNP formulations induced significant cytotoxicity at an N/P ratio of 10 (*p* > 0.05), indicating excellent biocompatibility under these conditions (Figure 4D).

### Generalizability of Surface-Modified LNPs-Based mRNA Delivery

Next, we further evaluated the versatility and generalizability of our LNPs-based mRNA delivery platform. We tested a second commercially available reporter mRNA encoding EGFP.^40^ We synthesized the EGFP mRNA-LNPs at an N/P ratio of 10 and modified the nanoparticle surface with either HEP or PEG polymers (Figure S5). The resulting HDD and PDI exhibited consistent trends as observed for firefly luciferase mRNA-LNPs (Figure S5A). Using RiboGreen-based assays, we determined >90% EE (Figure S5B). Importantly, XTT-based cell viability assays confirmed that EGFP mRNA-LNPs at all tested mRNA amounts, regardless of surface modification, did not induce significant cytotoxicity (*p* > 0.05; Figure S5C), while the LNPs number concentrations determined by FFF-MALS remained similar to the firefly luciferase mRNA-LNPs (~6 × 10^10^ LNPs/mL for both HEP- and PEG-modified LNPs; Figure S5D).

**Figure 5.**
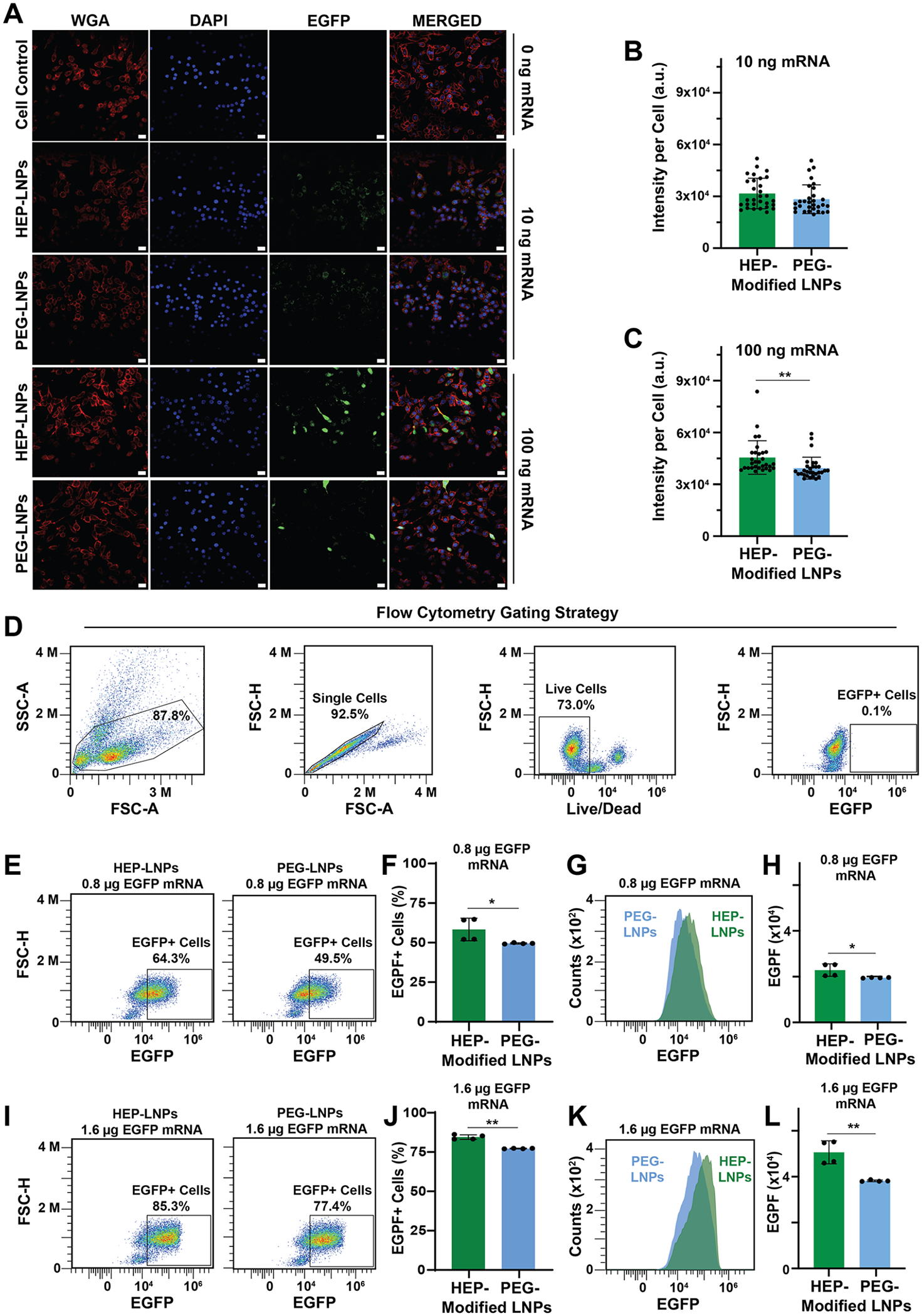
Cellular transfection efficiency of surface-modified EGFP mRNA-LNPs. (A) CLSM images of RAW264.7 murine macrophage cells after 24-h incubation with HEP- or PEG-coated mRNA-LNPs. Scale bar: 20 *μ*m. (B and C) CLSM image quantification results of EGFP fluorescence intensity using ImageJ, measured as integrated density within regions of interest drawn around cell membranes. Data are presented as mean ± standard deviation (*n* = 30, *p < 0.05, **p < 0.01, *t* test). (D) Flow cytometry gating strategy. (E–L) Comparison of the EGFP expressions in RAW264.7 cells transfected with HEP- or PEG-coated EGFP mRNA-LNPs at two different mRNA doses, i.e., 0.8 *μ*g or 1.6 *μ*g of EGFP mRNA. (E and I) Representative flow cytometry plots showing EGFP^+^ cell populations (boxed). (F and J) Bar graphs depicting the percentage of EGFP^+^ cells (mean ± standard deviation, *n* = 4, *p < 0.05, **p < 0.01, *t* test). (G and K) Overlaid histograms comparing the EGFP signal distributions between HEP- and PEG-modified mRNA-LNPs groups. (H and L) Bar graphs showing the mean EGFP intensity per cell population (mean ± standard deviation, *n* = 4, *p < 0.5, **p < 0.01, *t* test).

To directly visualize EGFP mRNA transfection of cells, we used CLSM imaging to detect the green fluorescence of EGFP to monitor transgene expression (Figure 5A).^38^ RAW264.7 murine macrophages were incubated for 24 h with EGFP mRNA-LNPs delivering 10 ng (40 ng/mL) or 100 ng (400 ng/mL) of mRNA per group. Robust green fluorescence signals were observed in both HEP- and PEG-modified mRNA-LNPs groups, confirming successful intracellular delivery and expression of EGFP (Figure 5A). We quantified EGFP signal intensity using ImageJ and observed comparable fluorescence in the HEP-modified and PEG-modified mRNA-LNPs groups (Figures 5B,C). As expected, higher mRNA concentrations produced stronger fluorescence signals. In the 100 ng EGFP mRNA group, HEP-coated mRNA-LNPs exhibited superior transfection performance compared to PEG-coated counterparts, yielding an average signal intensity ~13% higher than that of the PEG group.

To evaluate the mRNA transfection efficiencies of HEP- and PEG-modified mRNA-LNPs at the single-cell level, we used flow cytometry (Figure 5D–L).^41^ We incubated RAW264.7 murine macrophages with the corresponding EGFP mRNA-LNPs for 24 h. At a dose of 0.8 *μ*g (400 ng/mL) or 1.6 *μ*g (800 ng/mL) of EGFP mRNA, the HEP-LNP group yielded a higher percentage of EGFP-positive cells compared to the PEG-LNP group (parts E,F and I,J of Figure 5). The HEP-coated mRNA-LNP groups exhibited higher levels of EGFP-positive live cells compared to the PEG-coated counterparts (0.8 *μ*g, +14.8%; 1.6 *μ*g, +7.9%) (Figure 5G,K). In addition, the mean fluorescence intensity per cell was consistently greater in the HEP-LNP groups (Figure 5H,L). Notably, at the 1.6 *μ*g mRNA dose, the mean EGFP fluorescence intensity in the HEP-LNP group was ~33% higher than that of PEG-LNPs (Figure 5L), indicating enhanced transfection efficiency. These findings suggest that HEP surface modification improves the mRNA effectiveness, particularly at higher mRNA delivery doses.

The above findings suggest that, beyond luciferase mRNA, EGFP mRNA-LNPs were also successfully synthesized with controlled nanoparticle size, colloidal stability, mRNA encapsulation efficiency, and transfection performance, demonstrating the generalizability of our mRNA-LNPs formulation strategy. Consistent with the luciferase results, HEP-coated mRNA-LNPs achieved higher mRNA transfection efficiency than their PEGylated counterparts. The HEP surface modification further increased mRNA effectiveness, particularly at higher mRNA delivery doses, highlighting its potential as a biocompatible alternative to PEG for improved mRNA efficacy.

### *In Vivo* Efficacy and Safety Evaluation

**Figure 6.**
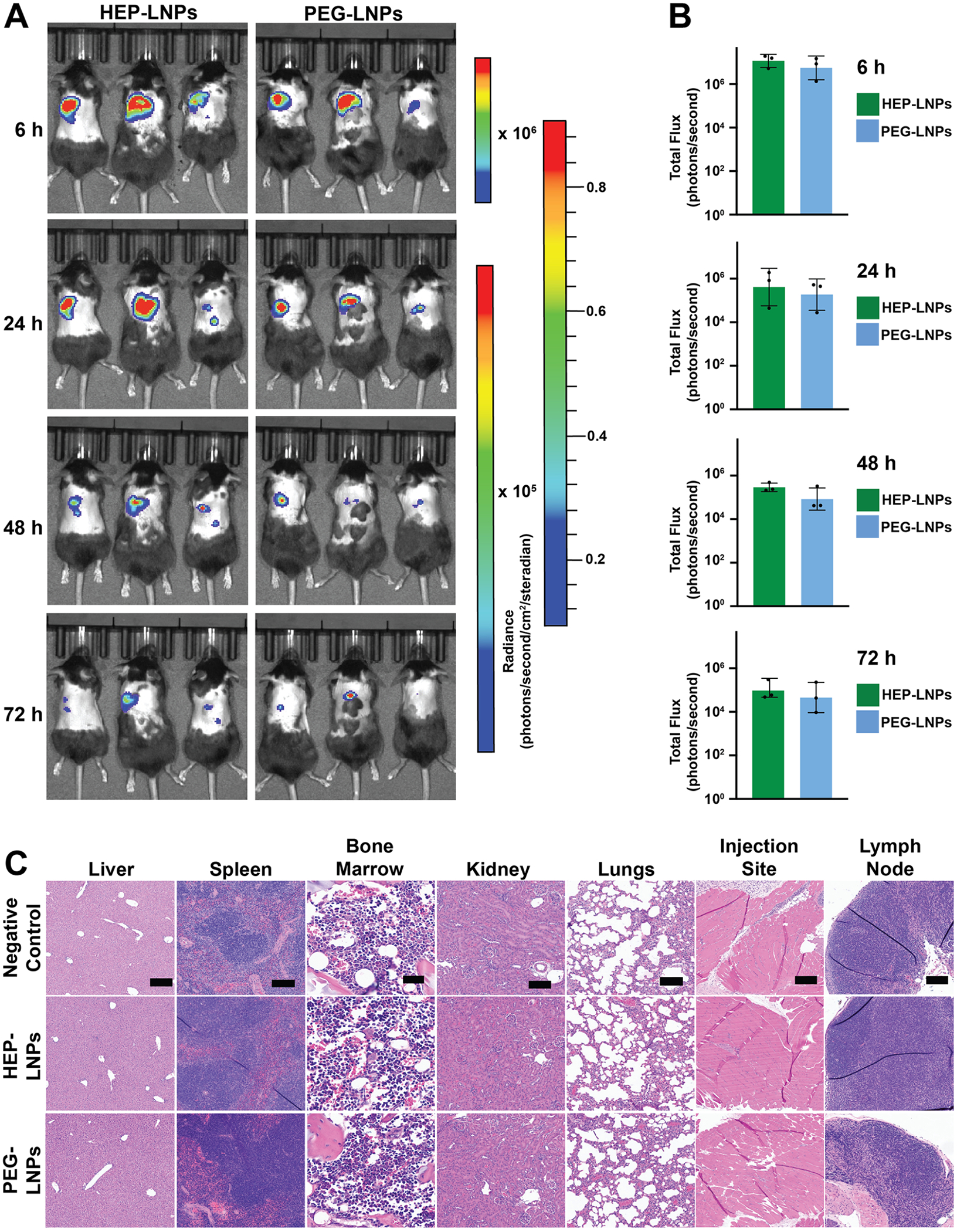
*In vivo* bioluminescence and histopathology of C57BL/6 mice following subcutaneous administration of HEP- or PEG-coated firefly luciferase mRNA-LNPs. C57BL/6 female mice (*n* = 3/group) were subcutaneously injected with 2 *μ*g of firefly luciferase mRNA-LNPs coated with either HEP or PEG in 100 *μ*L of 1× PBS. Whole-body bioluminescence was monitored at 6, 24, 48, and 72 h postinjection using the IVIS SpectrumCT imaging system, with representative images (A) and quantified total luminescent flux (B) shown. The color scales depict the corresponding radiance signal intensities for the different time points. Image acquisition and analysis were performed using Living Image software. (C) Representative H&Estained sections of major organs collected after two subcutaneous injections, administered 2 weeks apart. A total of 4 *μ*g of firefly luciferase mRNA-LNPs (2 *μ*g, 100 *μ*L, 1× PBS, each), coated with HEP, PEG, or PBS as a negative control, was administered. Black scale bars: liver, 200 *μ*m; spleen, 100 *μ*m; bone marrow, 35 *μ*m; kidney, 100 *μ*m; lung, 100 *μ*m; injection site, 200 *μ*m; and lymph node, 100 *μ*m.

We then formulated luciferase mRNA-LNPs at an N/P ratio of 5 for *in vivo* safety and efficacy evaluation. For *in vivo* experiments, an N/P ratio of 5 was selected to reduce total lipid exposure and improve tolerability, as lower N/P ratios are generally preferred for *in vivo* administration.^38^ As in previous experiments, we modified the LNP surface with either HEP or PEG polymers (Figure S6). To evaluate the *in vivo* efficacy and biodistribution of HEP- and PEG-modified firefly luciferase mRNA-LNPs, we administered 2 *μ*g of mRNA doses via subcutaneous injection in mice (Figure 6A).^42^ We monitored the bioluminescence signals at 6, 24, 48, and 72 h postinjection using an IVIS imaging system. The strongest bioluminescence signal was localized at the injection site 6 h postinjection and gradually decreased over time, consistent with the transient expression profile of mRNA agents (Figure 6A).^43^ Across four time points, HEP-modified mRNA-LNPs consistently tended toward higher bioluminescence signals compared to the PEG-modified mRNA-LNPs counterparts (Figure 6B).

To assess the biodistribution, we performed *ex vivo* IVIS imaging of major organs following subcutaneous administration. Notably, neither HEP- nor PEG-modified LNPs exhibited detectable luminescent signals in the liver, spleen, or other major organs (Figure S7), indicating minimal systemic distribution under these conditions.

This localized expression profile is consistent with prior reports on subcutaneous and intradermal nucleic acid delivery systems. These studies confirmed that uptake and transfection were largely restricted to the injection site and draining lymphatic tissues, with primary involvement of local antigen-presenting cells, such as dendritic cells and macrophages, rather than distant organs.^44^ Given the absence of detectable hepatic luminescence signals using our subcutaneous delivery condition, interactions between hepatic cells and our LNPs systems may be minimal.^2^ Instead, both of our tested LNPs formulations appeared to function within a localized subcutaneous area at the injection site, where nanoparticle–cell interactions may be dominated by resident immune cell populations. While IVIS imaging may not detect low levels of LNPs organ accumulation, alternative approaches, such as radiolabeling, qPCR-based biodistribution analysis, or histological and cellular colocalization studies, could be explored in future work.^45,46^ We further observed no significant changes in mouse body weight for all treatment groups (Figure S8).

To further interpret the cellular basis of the observed *in vivo* expression following subcutaneous administration, we considered the cell types most likely to be transfected based on prior literature. The *in vitro* studies in this work were performed on macrophages to model professional phagocytic cells, which are highly relevant to subcutaneous delivery.^44^ Following subcutaneous administration, it has been reported that nanoparticle–cell interactions occur predominantly in local cell populations at the injection site and draining lymphatics. The primary cell types typically include dendritic cells and macrophages, which play central roles in antigen presentation and immune activation.^47^

Consistent with prior reports on subcutaneous and intradermal nucleic acid delivery systems, antigen-presenting cells, such as dermal dendritic cells and monocyte-derived macrophages, represent primary targets, while fibroblasts and other stromal cells may contribute to local expression to a lesser extent.^48^ In contrast, intravenously administered LNPs often exhibit strong liver accumulation and often interact with hepatocytes, liver-resident macrophages (Kupffer cells), and other cells of the mononuclear phagocyte system.^2^

While we did not focus on evaluating cell-type-specific transfection in this study, our use of macrophages provided a relevant *in vitro* approximation of a primary *in vivo* target cell population under subcutaneous administration conditions.^32^

To evaluate the *in vivo* biocompatibility of the formulations, we administered the HEP- or PEG-modified firefly luciferase mRNA-LNPs to mice via two subcutaneous injections. The injections were performed 2 weeks apart. We performed histopathological analysis 2 days after the second injection and compared the results with negative control groups to assess potential tissue toxicity and local inflammatory responses.^49^

We performed histopathological analysis 2 days after the booster administration to evaluate acute tissue responses following repeated exposure. We selected this time point to capture potential acute tissue changes while minimizing long-term tissue remodeling effects.^49^ We did not collect blood samples for cytokine or antibody analysis, and therefore, immune activation was not directly assessed in this study.

We evaluated major organs, including the liver, spleen, tibial bone marrow, kidney, lungs, injection site, and left axillary draining lymph node.^49^ Hematoxylin and eosin (H&E) staining did not reveal any detectable abnormalities in the liver, spleen, bone marrow, kidney and axillary draining lymph node of any treatment group (Figure 6C and Table S3), indicating minimal systemic or hematopoietic toxicity following subcutaneous administration of HEP- or PEG-coated mRNA-LNP formulations. In the lungs, subsets of mice exhibited interstitial widening involving ~40–70% of the tissue, accompanied by cellular infiltrates and mild vascular congestion. However, similar findings were also observed in the negative control group, suggesting these changes were not specific to the treatments. At the injection site, localized changes included scattered acute inflammatory cells, focal fat necrosis, and inflammatory cell infiltration surrounding necrotic areas, although some samples showed no abnormalities. Following two subcutaneous administrations, HEP-coated LNPs showed no evidence of increased tissue damage, inflammatory infiltration, or organspecific pathology compared to PEG-coated controls.

Compared with PEG-modified LNPs, which are widely used in nanoparticle formulations to enhance colloidal stability, our HEP-modified LNPs demonstrated comparable physicochemical properties and mRNA delivery efficiency in both *in vitro* and *in vivo* models. Prior studies have shown that surface chemistry plays a critical role in nanoparticle biodistribution and immune recognition,^9,19,20^ and our results suggest that HEP can achieve similar functional performance under subcutaneous delivery conditions. However, future studies that focus on comparative evaluations of pharmacokinetics and immune responses will be valuable for fully establishing differences between these surface modification strategies.

A key consideration in evaluating alternatives to PEG is their immunogenicity profile, including the potential to induce antipolymer antibodies, complement activation, and inflammatory cytokine responses.^13^ In the present study, we did not directly assess these parameters. We evaluated the *in vivo* performance of luciferase mRNA-LNPs following a single subcutaneous administration, while performing safety assessment and histopathological analysis under a two-dose regimen.

Across these studies, we observed no evidence of overt toxicity or tissue damage, and histological examination indicated overall tissue integrity and safety under the tested conditions. The comparable *in vivo* expression profiles observed between HEP-and PEG-modified LNPs suggest that the HEP polysaccharide functionalization does not introduce immediate functional limitations in the context of subcutaneous delivery. Given that PEGylated nanocarriers have been associated with anti-PEG antibody formation and accelerated blood clearance upon repeated administration, the use of a naturally derived polysaccharide, such as HEP, may reduce immunological risk.^13,17,18^

Notably, prior studies of HEP-coated liposomal systems have reported minimal induction of anti-HEP IgG or IgM antibodies *in vivo*, suggesting a potentially favorable immunological profile of HEP-based surface modifications. Future studies will focus on a comprehensive evaluation of immunogenicity, including antipolymer antibody production, complement activation, and cytokine responses, to directly compare the immune profiles of HEP- and PEG-modified LNPs and fully establish their safety and translational potential.

### Comparison with Previously Reported LNP Surface Modifications

In our work, across all tested formulations, regardless of the N/P ratio (100, 10, or 5) or mRNA type (luciferase or EGFP), LNPs coated with either HEP or PEG exhibited comparable physicochemical characteristics. Both coatings increased HDD values by ~20–50 nm compared to uncoated LNPs. All formulations displayed low PDI (<0.2), high encapsulation efficiency (~90%), and comparable particle concentrations (~1 × 10^11^ nanoparticles mL^−1^) across groups. These findings collectively confirm that our synthesis approach yields structurally consistent and robust mRNA-LNPs across different N/P ratios and mRNA cargos. Both luciferase and EGFP mRNA were successfully delivered using HEP- and PEG-modified LNPs *in vitro* or *in vivo*, achieving efficient transfection without noticeable cytotoxicity.

Our HEP-mRNA-LNPs tended to produce higher transfection efficiency than PEG-mRNA-LNPs, particularly at higher mRNA input. Histopathological evaluation revealed no treatment-specific abnormalities in major visceral organs following repeated subcutaneous administration, indicating that HEP-mRNA-LNPs were well tolerated *in vivo* with no evidence of systemic toxicity. Since the HEP coating is applied after nanoparticle formation, the strategy is independent of the encapsulated mRNA sequence or reporter, as demonstrated using both luciferase and EGFP mRNA. From a translational perspective, the post-formulation coating strategy is compatible with scalable manufacturing and quality control, as it avoids changes to lipid composition, ionizable lipid chemistry, or formulation parameters that are tightly regulated in current LNP production pipelines.

In Table 1, we provide a broader comparison of our HEP coating approach with other PEG alternatives across the literature. We summarized examples of recent strategies used to overcome limitations of PEGylated LNPs systems by modifying or replacing PEG-lipids. Researchers have explored a wide range of alternative surface materials, including polysaccharide-based, polymer-based, and zwitterionic lipid-based PEG-replacement strategies, to enhance nanoparticle stability, reduce immunogenicity, and improve tissue-specific mRNA delivery. In parallel, PEG-modification strategies employing cleavable or degradable linkers have been developed to preserve colloidal stability while promoting improved cellular interactions and uptake.

To contextualize these prior advances, we compared our HEP-coated mRNA-LNP formulation with representative PEG-replacement systems reported in the literature. We summarized key formulation parameters, including nanoparticle size, PDI, composition setup, and encapsulation efficiency, alongside efficacy readouts, toxicity profiles, and reported limitations. This comparative analysis may guide readers in selecting potential PEG alternatives for nanomedicine applications and highlights the reproducible, robust synthesis of HEP-coated mRNA-LNPs with excellent physicochemical properties. The HEP-coated mRNA-LNPs exhibit good transfection efficiency and excellent biocompatibility, underscoring their potential as a practical, scalable alternative to PEG-modified LNPs.

## CONCLUSIONS

In summary, our study demonstrates that the HEP-coated mRNA-LNP platform presented here is a reliable, robust, and reproducible surface-engineering strategy. Although the increase in mRNA expression relative to PEG-coated LNPs is modest, achieving comparable delivery performance without PEG represents a meaningful advance, given growing concerns about PEG immunogenicity and regulatory complexity. Across a broad range of N/P ratios, HEP-coated mRNA-LNPs exhibited stable nanoparticle size, narrow polydispersity, and efficient mRNA encapsulation.

The HEP polysaccharide polymers proved to be an effective and biocompatible alternative to PEG for LNP surface engineering. The HEP-mRNA-LNPs achieved transfection efficiency comparable to or higher than that of their PEGylated counterparts, both *in vitro* and *in vivo*, while inducing minimal tissue injury and negligible immune activation. *In vivo* bioluminescence imaging further confirmed localized and transient luciferase expression with no detectable off-target accumulation. Together, these results highlight the potential of HEP to replace PEG in mRNA-LNP delivery systems in applications including vaccination, gene therapy, and cancer immunotherapy.

Future studies might expand upon these findings to further optimize the HEP-coated mRNA-LNP design. First, different bioconjugation chemistries, including maleimide or click-based linkers, could be explored to determine how the mode of HEP attachment affects nanoparticle uptake and intracellular trafficking. Second, the molecular weight of HEP may play a key role in delivery efficiency. Investigating a wider range of polymer sizes beyond the 13-kDa OPSS-HEP used here will clarify size-dependent effects at both cellular and organismal levels. Third, assessing the HEP coating of LNPs across various ionizable lipid candidates could reveal formulation-specific benefits to enhance functional mRNA delivery, efficacy, and safety.

In addition to the future studies mentioned above, immunological responses to repeated administration represent an important consideration for LNP-based systems.^65^ The potential for accelerated blood clearance (ABC) and altered pharmacokinetics upon repeated administration is an important consideration for PEGylated LNP systems.^32^ In the present study, we did not perform a systematic evaluation of pharmacokinetics or repeat dosing effects, and therefore cannot directly assess whether HEP-modified LNPs mitigate ABC phenomena relative to PEGylated formulations. Nevertheless, given the well-documented association of PEG with anti-PEG antibody formation and subsequent ABC responses, the use of a naturally derived polysaccharide such as HEP may represent a promising alternative to reduce these effects.^9,17^ While our observations from single-dose studies suggest comparable *in vivo* performance between HEP- and PEG-modified LNPs, a comprehensive investigation of pharmacokinetics, repeat dosing, and immune responses is warranted in future studies to determine whether HEP functionalization confers advantages in this context.^65^

While this study establishes HEP polysaccharides as a PEG-free surface modification that preserves mRNA delivery performance and repeated-dose tolerability, future studies will be required to systematically evaluate polymer-specific immune responses, including anti-HEP antibodies, complement activation, pharmacokinetics, accelerated blood clearance, and potential cross-reactivity with pre-existing anti-PEG antibodies.

A comprehensive understanding of these parameters will accelerate the development of safer, biodegradable, immunologically inert, and effective mRNA-LNP platforms. Our results suggest that HEP-based LNP surface engineering strategies hold strong potential to advance next-generation PEG-free nanomedicines.

## EXPERIMENTAL SECTION

### General Materials

M5–0.8 × 1 m threaded rod (Fabory, M20230.050.1000, Norman, OK, United States); M5 Nut (Ace Hardware, 5165774, Moore, OK, United States); 5 × 5 mm shaft coupler (Befenybay, BE-032–7-fba, Norman, OK, United States); Ender3 Kit (Amazon, Norman, OK, United States); BD PrecisionGlide needles (Fisher Scientific, BD305122, Houston, TX, United States); Amicon Ultra-4 centrifugal filter unit, 100-kDa molecular weight cutoff (MWCO; Millipore Sigma, UFC 10024, St. Louis, MO, United States); Amicon Ultra-0.5 centrifugal filter unit, 100-kDa MWCO (Millipore Sigma, UFC510096, St. Louis, MO, United States); 1.6 mm OD T-mixer (Idex Health and Science, P-712, Carlsbad, CA, United States); PEEK Cross mixer, 1.6 mm OD tubing (Restek, 27722, Bellefonte, PA, United States); 25G needles (Fisher Scientific, Houston, TX, United States); 305124, 10 mL Luerlock syringe (Fisher Scientific, B302995, Houston, TX, United States); BD Slip Tip 1 mL syringe (Fisher Scientific, BD309659, Houston, TX, United States).

### LNP Materials

(6*Z*,9*Z*,28*Z*,31*Z*)-Heptatriaconta-6,9,28,31-tetraen-19-yl 4-(dimethylamino)butanoate (DLINMC3-DMA; Medkoo Bioscienes, 555308, Morrisville, NC, United States); 1,2 distearyol-*sn*-glycero-3-phosphocholine (DSPC; Avanti Polar Lipids, 850365, Alabaster, AL, United States); cholesterol (Avanti Polar Lipids, LM4100, Alabaster, AL, United States); 16:0 Ptd Thioethanol (Avanti Lipids, 880151, Alabaster, AL, United States); mPEG-OPSS MW 10 kDa (Laysan Bio, 162–32, Arab, AL, United States). HEP and OPSS-HEP were synthesized and characterized in house, and the general conjugation processes followed our published methods here.^17,18^ Citrate buffer (pH 4.0, 100 mM) (Fisher Scientific, Q2444, Houston, TX, United States); RiboGreen Quantification Kit (Thermofisher, R11490, Houston, TX, United States); Spectra/Por 2 Trial Kit, 12–14 kDa (Repligen, 132678T, Boston, MA, United States); Five EZ Cap Firefly Luciferase mRNA (APExBIO, R1018, Houston, TX, United States); EZ Cap EGFP mRNA (5-moUTP) (APExBIO, R1016, Houston, TX, United States); Luciferase Assay System (Promega Products, E1501, Madison, WI, United States); D-luciferin potassium salt [Gold Biotechnology (U.S. Registration No 3,257,927)].

### Cell Culture Materials

RAW 264.7 mouse macrophages (ATCC, TIB-71, Manassas, VA, United States); Dulbecco’s modified Eagle’s medium (DMEM), high glucose, pyruvate (Thermo Fisher, 11995065, Houston, TX, United States); fetal bovine serum (FBS; Thermo Fisher, 16000044, Houston, TX, United States); penicillin–streptomycin (Thermo Fisher, 15-140-122, Houston, TX, United States); paraformaldehyde solution (PFA), 4% in PBS (Thermo Fisher, J19943K2, Houston, TX, United States); wheat germ agglutinin (WGA), CF633 conjugate (Biotium, 29024, Fremont, CA, United States); NucBlue Fixed Cell ReadyProbes reagent (DAPI) (Thermo Fisher, R37606, Houston, TX, United States); 18 mm round coverslips #1 (VWR, 16004–300, Missouri City, TX, United States); #1.5H glass bottom dishes (Fisher Scientific, 5003050807, Houston, TX, United States); 12-well cell culture plate (VWR, 10062–894, Missouri City, TX, United States); *μ*-Slide 8-well high glass bottom (ibidi, Cat. No. 980807, Fitchburg, WI, United States); Pierce 96-well polystyrene plates, white opaque (Thermo Fisher, 15042, Houston, TX, United States).

### Instruments

DLS (Malvern Zetasizer Nano ZS, Enigma Business Park, Malvern, U.K.); FFF-MALS (Wyatt Technology, Santa Barbara, CA, United States); centrifuge (Thermo Scientific, Heraeus Multifuge X3R, Houston, TX, United States); plate reader (Aglient, BioTek Synergy Neo2, Santa Clara, CA, United States); Andor BC43 Benchtop confocal microscope (Andor, Belfast, U.K.); Cytek Northern Lights flow cytometry (Cytek Biosciences), data analyzed using FlowJo v10.7.1.; PerkinElmer IVIS Spectrum CT (PerkinElmer, Inc., Waltham, MA, United States).

## METHODS

### LNPs Preparation and Surface Modification

The LNPs were prepared using fluidic mixing, following a procedure by Young et al.^21^ Briefly, an ethanolic lipid phase with an aqueous mRNA solution in citrate buffer (pH 4.0–4.5). The lipid composition [DLIN-MC3-DMA ((10*Z*,13*Z*)-1-(9*Z*,12*Z*)-9,12-octadecadien-1-yl10,13-nonadeca-dien-1-yl ester)/DSPC/cholesterol/thiol-lipid, 50:10:38.5:1.5 mol %] and total lipid concentration (3.2 mM) were adapted from the literature.^22^ The flow-rate ratio (aqueous/organic) was set to 7 to control the particle size. After nanoparticle formation, 250 *μ*L of 10 mM HEPES buffer (pH 12) was added to 4 mL of LNPs suspension to adjust the pH to ~7.0. The surface modification was performed via thiol disulfide exchange between thiol-containing lipids and OPSS-HEP or OPSS-PEG at defined molar ratios, enabling surface functionalization without perturbing the LNP core structure. The LNPs were dialyzed against PBS to remove ethanol and equilibrate pH. Briefly, the LNPs suspension was transferred to dialysis tubing (MWCO = 12–14 kDa) and dialyzed against 1× PBS (pH 7.4) at 4°C with gentle stirring. A buffer volume at least 100-fold in excess of the sample was used, and the dialysis buffer was replaced 2–3 times over 12–24 h to ensure complete removal of ethanol and equilibration to physiological conditions.

### Physicochemical Characterization

The HDD, PDI, and *ζ* potential were measured with a Malvern ZetaSizer Nano ZS instrument. The nanoparticle number concentration and size distributions were determined by FFF-MALS. The mRNA encapsulation efficiency was quantified using the commonly used RiboGreen assay (Thermofisher, R11490, Houston, TX, United States).

### Quantification of mRNA Encapsulation Efficiency by Reverse Transcription Quantitative PCR (RT-qPCR)

The encapsulation efficiency of luciferase mRNA in LNPs was determined using an RNase protection assay coupled with RT-qPCR. Briefly, mRNA-LNP samples were treated with RNase to degrade unencapsulated mRNA, followed by quenching and detergent-mediated lysis. Encapsulated mRNA was purified using a silica membrane spin column and reverse transcribed into cDNA using the RevertAid First Strand cDNA Synthesis Kit (Thermo Scientific, Cat. No. K16215) with gene-specific primers (TaqMan Gene Expression Assay, Thermo Scientific). qPCR was performed using Phusion Plus Green PCR Master Mix (Thermo Scientific, Cat. No. F631L) on a CFX Opus 96 (Bio-Rad) system under standard cycling conditions. Absolute mRNA quantification was obtained using standard curves generated from serial dilutions of naked mRNA processed in parallel. Equation 1 was used to calculate the EE%:

(1) $$\text{EE}\%=\frac{\text{mRNAprotected}}{\text{mRNAtotal}}\times 100\%$$

All measurements were performed in triplicate, and data were analyzed using Bio-Rad CFX Maestro 2.3 software.

### PAGE Analysis of Surface-Conjugated HEP

Surface-conjugated HEP on LNPs was analyzed by PAGE. Samples (2 *μ*g per lane) of HEP-NH_2_, HEP-OPSS, HEP-LNP, and dithiothreitol (DTT)-treated HEP-LNP were resolved on 8% polyacrylamide gels in 1× TBE buffer. DTT treatment was used to cleave disulfide linkages and release surface-bound HEP. Electrophoresis was performed at 250 V for 25 min. Gels were stained with Alcian Blue to visualize sulfated polysaccharides. The HEP content was quantified by comparison to HEP-NH_2_ standards.

### Calculation of HEP Molecules per LNP

Based on gel quantification, the measured HEP concentration was 0.12 *μ*g of HEP per *μ*L of LNP suspension. The LNP concentration was determined using FFF-MALS.

The calculated mass of HEP per individual LNP was 1.2 × 10^−9^ ng per LNP (or 1.2× 10^−18^ g per LNP)

Given the molecular weight of HEP (13 kDa = 13000 g/mol), the number of moles of HEP per LNP was calculated using eq 2:

(2) $$\frac{1.2\;\times \;10^{-18}\;\text{g}}{13000\;\text{g/mol}}=9.23\times 10^{-23}\;\text{mol}$$

Converting moles to molecules using Avogadro’s number (6.022 × 10^23^ mol^−1^) according to eq 3:

(3) $$9.23\times {\text{10}}^{-23}\times 6.022\times 10^{23}\approx 56\;\text{molecules}\;\text{per}\;\text{LNP}$$

Thus, each LNP contains approximately 56 HEP molecules.

### Surface Density of HEP

Assuming a spherical LNP with radius *r* = 80 nm and eq 4, the surface area is

(4) $$A=4\pi r^{2}=4\pi {\left(80\;\text{nm}\right)}^{2}\approx 8.04\times 10^{4}\;{\text{nm}}^{2}$$

According to eq 5, the surface density of HEP is therefore

(5) $$\frac{56}{8.04\times 10^{4}\;{\text{nm}}^{2}}\approx 7.0\times 10^{-4}\;\text{HEP}\;\text{per}\;{\text{nm}}^{2}$$

This corresponds to approximately

$$\sim 1\;\text{HEP}\;\text{molecule}\;\text{per}\;1400\;{\text{nm}}^{2}$$

### *In Vitro* Transfection and Cytotoxicity

A total of 10000 RAW 264.7 murine macrophage cells were seeded per well into a white-walled, clear-bottom 96-well plate, using 100 *μ*L of a complete cell culture medium. The medium consisted of DMEM supplemented with 10% FBS and 1% (v/v) penicillin–streptomycin. The cells were allowed to adhere overnight in a humidified 37 °C incubator with 5% CO_2_.

The following day, mRNA-loaded LNPs were diluted in a fresh complete medium to achieve the desired dose in 100 *μ*L per well. The old medium was gently removed from each well, and cells were treated with either the diluted mRNA-LNP formulations or a complete medium alone (negative control). The treated plate was returned to the incubator and incubated for 24 h.

To evaluate transfection efficiency, a luciferase assay was performed. The luciferase reagent and 1× cell lysis buffer were prepared following the manufacturer’s protocol. The 96-well plate was transferred to a biosafety cabinet. Media were aspirated from each well, and cells were washed once with 1× PBS. Then, 20 *μ*L of 1× lysis buffer was added to each well, followed by 100 *μ*L of the luciferase assay reagent. The plate was protected from light and immediately analyzed using a BioTek Synergy Neo2Multi-Mode plate reader to measure bioluminescence. All luminescence values were exported and analyzed using GraphPad Prism. Transfection efficiency was quantified based on normalized luminescence intensity.

Explanation: At an N/P ratio of 10, the total mRNA input used in *in vitro* transfection experiments varied depending on the assay format and culture scale. For transfection assays performed in 96-well plates, 5–40 ng luciferase mRNA per well was used with approximately 10,000 cells seeded per well, whereas larger culture formats such as 12-well plates required higher total EGFP mRNA input (0.8–1.6 *μ*g per well) to maintain comparable exposure conditions for approximately 200,000 cells per well. Accordingly, the increase in total mRNA input reflects differences in culture scale rather than changes in effective dosing conditions on a per-cell basis. mRNA input is therefore reported as the total amount per well based on experimental format, and, where applicable, corresponding concentrations are also provided to facilitate comparison across studies.

### Confocal Laser Scanning Microscopy (CLSM)

The EGFP expression, cellular uptake, and intracellular distribution were analyzed by CLSM using an Andor BC43 spinning disk CLSM instrument, following staining of nuclei and plasma membranes. For DiO-labeled LNPs, Vybrant DiO Cell-Labeling Solution (Thermo Fisher Scientific, V22886, Eugene, OR, United States) was dissolved in 100% ethanol at 0.044 mg/mL and incorporated into the lipid mixture prior to microfluidic mixing. Dialysis was performed overnight in 1× PBS to remove unincorporated dye.

### Flow Cytometry

RAW 264.7 cells were seeded in 12-well plates at a density of 2 × 10^5^ cells per well in complete medium and incubated overnight at 37 °C with 5% CO_2_ to allow cell adhesion. The next day, cells were treated with 0.8 mg or 1.6 mg of EGFP mRNA encapsulated in LNPs surface-modified with either HEP or PEG. As controls, a negative control group (untreated; no mRNA or transfection reagent) and a positive control group (transfected with 2 mg EGFP mRNA using Lipofectamine according to the manufacturer’s instructions) were included.

After a 24-h incubation, cells were washed once with 1× PBS, detached using a sterile cell scraper, and immediately transferred to ice to halt further endocytosis and exocytosis. All subsequent steps were performed on ice. Cells were pelleted by centrifugation, and the supernatant was removed. The pellets were washed once with ice-cold 1× PBS and centrifuged at 500 X g for 3 min. The resulting cell pellets were resuspended in 300 *μ*L PBS, and 5 *μ*L of ViaDye Red Fixable Viability Dye (Cytek, SKU R7–60008), prediluted 1:500, was added to each sample. Staining was performed on ice for 15 min to assess membrane integrity and viability. Following staining, cells were washed once more with PBS by centrifugation and resuspended in 300 *μ*L PBS for flow cytometry. Controls included unstained cells, single-stained cells (ViaDye Red only), and cells treated with LNPs but not stained, to facilitate spectral unmixing and compensation. Samples were analyzed using a Cytek Northern Lights spectral flow cytometer (Cytek Biosciences). A minimum of 20000 events was collected per sample. Data acquisition was followed by analysis using FlowJo software (v10.7.1).

### *In Vivo* Bioluminescence Imaging

Eight-week-old C57BL/6J mice (Jax #000664) were purchased from Jackson Laboratories. The investigators followed the “Guide for the Care and Use of Laboratory Animals” by the Committee on Care of Laboratory Animal Resources Commission on Life Sciences, National Research Council. The animal facilities at the University of Oklahoma are fully accredited by the American Association for Accreditation of Laboratory Animal Care (AAALAC). All studies were conducted using protocols approved by the University of Oklahoma IACUC (Protocol No. 2024–0301).

To evaluate the *in vivo* transfection efficiency of surface-modified LNPs, C57BL/6 mice (8 weeks old, *n* = 3 per group) were subcutaneously injected with 2.0 *μ*g of luciferase mRNA-LNPs coated with either HEP or PEG in 100 *μ*L of 1× PBS, delivered into the flank region under sterile conditions. Bioluminescence imaging was performed at 6, 24, 48, and 72 h postinjection using the IVIS SpectrumCT Imaging System (PerkinElmer). At each time point, mice were anesthetized with 2.5% isoflurane and injected subcutaneously with 150 mg/kg D-luciferin potassium salt (GoldBio, Cat No. LUCK).

After a 10 min incubation to stabilize the bioluminescent signal, whole-body luminescence signals were captured using the IVIS system with autoexposure settings, automatically adjusting exposure time, binning, and f/stop to optimize signal quality and avoid saturation. Bioluminescence intensity was visualized using a pseudocolor scale and quantified as total flux (photons/s) using Living Image Software (PerkinElmer). Regions of interest (ROIs) were drawn around the injection site to quantify signal intensity. All image acquisition and analysis were performed using Living Image Software.

### Histopathological Analysis

To evaluate the *in vivo* biocompatibility of the formulations, mice received two subcutaneous injections of HEP- or PEG-modified firefly luciferase mRNA-LNPs administered 2 weeks apart. Each injection contained 2 *μ*g of mRNA formulated in the respective LNPs. Two days after the second injection, the mice were euthanized via CO_2_ asphyxiation, and tissues were collected for histopathological analysis.

The major organs, including the liver, spleen, kidneys, lung, injection site, tibia bone, and draining lymph nodes, were collected at the designated study end points. Tissues were immediately fixed in 10% neutral-buffered formalin (VN-FF0266–1L, VWR, VION) for a minimum of 72 h, dehydrated, and embedded in paraffin. Bone specimens were subjected to decalcification by immersion in EDTA/sucrose solution (Decalcifying Solution, 1048C, Newcomer Supply) for 14 days prior to embedding.

Paraffin blocks were sectioned at 4–5 *μ*m using a rotary microtome and mounted on glass slides. For routine histopathology, sections were stained with H&E according to standard protocols. Slides were examined under light microscopy (Leica DM5000B or equivalent) by a board-certified pathologist blinded to treatment groups. Histological evaluation focused on necrosis, inflammation, fibrosis, immune cell infiltration, and other pathological alterations. Positive and negative controls were included in all staining procedures. Histopathological findings were recorded semiquantitatively as incidence and severity scores following established criteria. Data were summarized as incidence (*a*/*b*), where *a* represents the number of affected animals and *b* the total number examined. Representative images were acquired using a digital pathology scanner (Aperio AT2, Leica Biosystems) at 20× magnification.

### Statistical Analysis

Data are presented as mean ± standard deviation unless otherwise stated. Statistical significance was determined using GraphPad Prism, with *p* < 0.05 considered significant.

## Supplementary Material

supporting information

The Supporting Information is available free of charge at https://pubs.acs.org/doi/10.1021/acsami.6c05213.

RiboGreen assay workflow, quantification of surface-conjugated HEP on HEP-LNPs by PAGE, CLSM images, results from FFF-MALS and DLS analyses, *ex vivo* imaging of mouse organs, body weight monitoring, and histopathological evaluation (PDF)

## Figures and Tables

**Table 1.** Comparison of HEP-Coated mRNA-LNPs with PEG-Replacement Systems^a^

category	surface coating	LNP composition	LNP design features	LNP size/PDI	cargo and EE (%)	efficacy readout	toxicity/safety	limitations	ref
polysaccharides	HEP	MC3	HEP-coated LNPs	~160 nm	mRNA	luciferase bioluminescence	*in vitro* XTT cell viability	further investigation needed	this study
		DSPC		PDI 0.1	>90% total (Ribo-Green)	*in vitro* and *in vivo*	*in vivo* histopathology study		
		cholesterol			>80% functional (PCR)	EGFP fluorescence *in vitro*	biocompatible		
		thiol-lipid				HEP-coated mRNA-LNPs tend to be more effective than PEG-coated counterparts			
	chitosan	DOTAP	chitosan-coated LNPs	300 nm	mRNA	PVX1010/circ-mRNA *in vitro*	*in vitro* AlamarBlue	contains PEG	*Vaccines* (2024)^50^
		DOPC		PDI n/a	~80%	linear mRNA-LNP	cytotoxicity assay	postassembly electrostatic coating	
		cholesterol				*in vivo* antibody response (chitosan enhances mucosal uptake)	biocompatible		
		chitosan							
		DSPE-PEG2000							
	hyaluronic acid (HA)	DOTAP	post-assembly HA layer on liposome-mRNA core	140 nm	mRNA	luciferase bioluminescence	*in vitro* cytotoxicity assay	postassembly electrostatic coating	*J. Controlled Release* (2023)^51^
		DOPE		PDI 0.2	N/A	EGFP	biocompatible	no direct comparison with PEG-coated counterparts	
		PE-Rho				CD90.1 mRNA			
		PE-DTPA				*in vitro* and *in vivo*			
	HA	DODMA	HA-coated LNPs	~180 nm	siRNA/miRNA	siRNA/miRNA delivery	*in vitro* cytotoxicity assay	multistep LbL coating	*Biomaterials* (2023)^52^
		cholesterol		PDI 0.2	97%	*in vitro* and *in vivo*	biocompatible		
		DOPE							
	Pullulan shell (LNP concept)	LNP (core unspecified)	Pullulan-coated LNPs	N/A	N/A	myeloid cell targeting	N/A	not peer-reviewed	*United Immunity* (2024–25)^53^
polymers	Polysarcosine (pSar) lipid	ALC-0315/SM-102 DSPC	LNPs	100–250 nm	mRNA	Luc/hEPO mRNA	*in vivo* similar ALT and AST to PEG-LNPs	risk of immunogenicity	*Bioactive Materials* (2024)^54^
		cholesterol	PEG-lipid fully replaced with pSar-lipid	PDI 0.01–0.23	70–90%	C_2_C1_2_, Hep3B; *in vivo* IVIS + hEPO			
		DMG-pSar	DMG-pSar_25_						
	poly(2-oxazoline/oxazine) (POx/POz) lipid	MC3	LNPs	1100–50 nm	mRNA	SARS-CoV2 RBD-TM nanoluciferase mRNA (N/P 6)	*in vitro* cytotoxicity assay	no *in vivo* safety data	*Biomacromolecules* (2024)^55^
		DSPC	POx/POz-lipid replaces PEG-lipid	PDI 0.1–0.2	~95%	in RAW264.7 + *in vivo*	biocompatible		
		cholesterol							
		POx/POz-lipid							
	PMOx [poly(2-methyl-2-oxazoline)] lipid	ionizable lipid H	LNPs	60–80 nm	mRNA	Rabies G	*in vivo* ALT and AST	risk of complement activation^56^	*Front. Drug Delivery* (2024)^57^
		cholesterol	PEG-lipid replaced with PMOx-lipid	PDI 0.1–0.4	~95%	EGFP mRNA			
		DSPC				*in vitro* and *in vivo* cytokines/T-cells			
		PMOz-DM-amide							
	linear polyglycerol (lPG) lipid	DSPC	LNPs	220–260 nm	mRNA	EGFP mRNA	*in vitro* Cytotoxicity Assay	limited *in vivo* data	*Macromol. Rapid Commun*. (2025)^58^
		linear polyglycerol (lPG) lipid	PEG-lipid replaced with lPG-lipid	PDI 0.11–0.17	78–93%	*in vitro* HepG2	biocompatible		
							*in vivo* low anti-PEG IgG reactivity		
zwitterionic lipid	pyridine carboxybetaine (PyCB) ionizable lipid	ALC-0315	LNPs	200–400 nm	mRNA	luciferase bioluminescence	*in vivo* ALT and AST	weakens at low pH or low ionic strength	*Sci. Adv*. (2025)^60^
		DSPC	cholesterol and PEG-lipid removed	PDI 0.2–0.3	90–95%	EGFP		increases in protein adsorption^59^	
		PyCB lipid	PyCB lipid provides stealth effect			Cre mRNA			
						spleen-specific translation			
						lower abc effect			
						repeat dose was tolerated			
	poly(carboxybetaine) (PCB) lipid	SM-102/MC3	LNPs	~100 nm	mRNA	EGFP	*in vivo* ALT and AST	pH sensitivity^59^	*Nai. Mater*. (2025)^61^
		cholesterol	PEG-lipid replaced with PCB-lipid	PDI 0.05–0.15	48–97%	Cas9	*ex vivo* cytotoxicity		
		DOPS				luciferase bioluminescence mRNA	biocompatible		
		PCB				better repeatability			
PEG-modification strategies	PEG2000-DMG (COVID-19 Vaccine Moderna)	SM-102	LNPs	~80–120 nm	mRNA	Phase 3 trial: 94.1% efficacy against symptomatic COVID-19 after 2 doses	common reactogenicity	PEG-induced anaphylaxis^16^	European Medicines Agency, Assessment Report – COVID-19 Vaccine Moderna, (2021)^62^
		DSPC	PEG-coated	PDI ≤ 0.2–0.25	N/A		rare PEG-linked anaphylaxis		
		cholesterol					rare myocarditis/pericarditis (mainly young males)		
		PEG2000-DMG					label updated		
							most cases resolve		
	PEG with different anchor-length (C14/C16/C18)	MC3	LNPs	<100 nm	siRNA	hepatic gene silencing	N/A	PEG-associated immunogenicity concerns^16^	*Mol. Ther. Nucleic Acids* (2013)^34^
		DSPC	formulated with fast-shedding C14/C16 or slow-shedding C18 PEG-lipid	PDI n/a	N/A	ED_50_			
		cholesterol							
		PEG-lipid							
	different ratios of DMG-PEG (C14) and DSPE-PEG (C18)	MC3	LNPs	~70 nm	mRNA	luciferase bioluminescence	PK focused	PEG-associated immunogenicity concerns	*Biomater. Sci*. (2023)^63^
		DSPC	varied DSPE-PEG (C18)/DMG-PEG (C14) ratios	PDI 0.06	~95%	Cy5-EGFP			
		cholesterol				siRNA (N/P 5.6)			
		DMG-PEG							
		DSPE-PEG							
	acid-degradable PEG-lipid (e.g., ADP-2k)	BP Lipid 312 (ionizable lipid 12)	LNPs	217–236 nm	CRISPR-Cas9 ribonucleoprotein	PCSK9 liver editing 31%	*in vitro* cytotoxicity assay	PEG-associated immunogenicity concerns	*Nat. Biotechnol*. (2024)^64^
		DOPE	pH-sensitive acetal PEG-lipid (ADP-2k, Pep-1*k*/2k)	PDI 0.10–0.13	~77%	lung editing 16–19%	biocompatible		
		cholesterol					*in vivo* ALT and AST		
		ADP-2k					immune responses		

a Abbreviations: Luc, luciferase; DOTAP, 1,2-dioleoyl-3-trimethylammonium-propane; DOPE, 1,2-dioleoyl-*sn*-glycero-3-phosphoethanolamine; PE-Rho, rhodamine-labeled phosphoethanolamine; PE-DTPA, diethylenetriaminepentaacetic acid-modified phosphoethanolamine; DODMA, 1,2-dioleyloxy-*N*,*N*-dimethyl-3-aminopropane; EPO, erythropoietin; ALT, alanine aminotransferase; AST, aspartate aminotransferase; LbL, layer-by-layer; MC3, DLin-MC3-DMG; RBD-TM, receptor-binding domain–transmembrane protein; ABC, accelerated blood clearance; Chol, cholesterol; PK, pharmacokinetics; ED50, median effective dose (50%).
