# Supplementary material for: Lipid Nanoparticle Surface Engineering with Heparosan Polysaccharides for Safe and Effective mRNA Delivery In Vitro and In Vivo
Source: ACS Appl Mater Interfaces. 2026 May 12;18(20):28536–55. doi: 10.1021/acsami.6c05213 (PMC13198436; doi:10.1021/acsami.6c05213)
Supplement: Supplementary file 1 [file am6c05213_si_001.pdf]

Supporting Information

Lipid Nanoparticle Surface Engineering with Heparosan Polysaccharides for Safe and Effective mRNA Delivery In Vitro and In Vivo

Yuxin He[1], Lin Wang[1], Rameswari Velayutham[2], Trisha I. Valerio[1], Samantha D. Ricketts[2], Yuanhong Sun[1], Anand C. Annan,[2] James Bowman[1], Thao Tran[1], Mobina Mohammadnejad[1], Kaili Liu[1], Wei R. Chen[1], Dixy E. Green[3], Kar-Ming Fung[2], Paul L. DeAngelis[3], Stefan Wilhelm[1,4,5,6,7,*]

[1] Stephenson School of Biomedical Engineering, University of Oklahoma, Norman, Oklahoma, 73019, USA

[2] Department of Pathology, University of Oklahoma Health Campus, Oklahoma City, Oklahoma, 73104, USA

[3] Department of Biochemistry and Physiology, University of Oklahoma Health Campus, Oklahoma City, Oklahoma, 73104, USA

[4] Institute for Biomedical Engineering, Science, and Technology (IBEST), University of Oklahoma, Norman, Oklahoma, 73019, USA

[5] Stephenson Cancer Center, University of Oklahoma, Oklahoma City, Oklahoma, 73104, USA

[6] Harold Hamm Diabetes Center, University of Oklahoma, Oklahoma City, Oklahoma, 73104, USA

[7] Materials Science and Engineering Program, University of Oklahoma, Norman, Oklahoma, 73019, USA

*Corresponding author:

Stefan Wilhelm, Ph.D.

Email: stefan.wilhelm@ou.edu ORCID: 0000-0003-2167-6221

Table of Contents

Supporting Figures..3

Figure S1: RiboGreen assay workflow for mRNA quantification..3

Figure S2: Quantification of surface-conjugated HEP on HEP-LNPs. 4

Figure S3: Confocal laser scanning microscopy (CLSM) demonstrating cell uptake of fluorescently labeled mRNA-LNP in RAW 264.7 macrophages..5

Figure S4: Reproducibility experiments to study the characterization and cell-level transfection of HEP- and PEG-modified firefly luciferase mRNA-LNPs with N/P ratio of 100...6

Table S1: Concentration of HEP- or PEG-modified mRNA-LNPs (N/P 100) quantified by field-flow fractionation multi-angle light scattering (FFF-MALS)......................................7

Table S2: Concentration of HEP- or PEG-modified mRNA-LNPs (N/P 10) quantified by FFF-MALS. 8

Figure S5: Characterization and *in vitro* cell viability assessment of HEP- and PEG-modified EGFP mRNA-LNPs with N/P ratio of 10..9

Figure S6: Characterization of HEP- and PEG-modified Luciferase mRNA-LNPs with N/P ratio of 5..10

Figure S7: *Ex vivo* bioluminescence imaging of organs from C57BL/6 mice 72 hours after treatment with HEP- or PEG-coated luciferase mRNA-LNPs. 11

Figure S8: Body weight monitoring of mice following repeated administration of firefly luciferase mRNA-LNPs...12

Table S3: Summary of Histopathological Findings..13

Supporting Figures

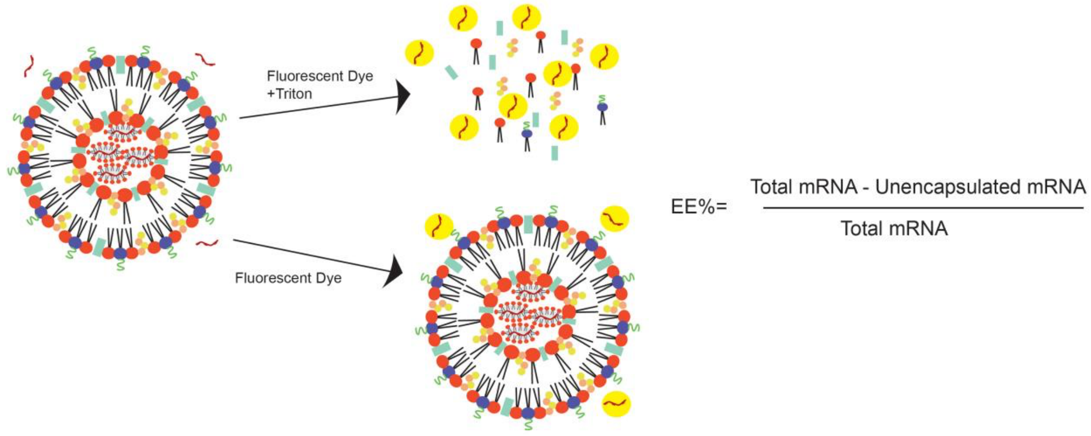

Figure S1: RiboGreen assay workflow for mRNA quantification. A simplified diagram illustrating the RiboGreen assay workflow to quantify mRNA concentration and encapsulation efficiency. LNPs are dispensed into wells of a well-plate with one set treated with RiboGreen reagent alone and the other set treated with RiboGreen and Triton. Triton, a detergent, lyses the LNPs, releasing encapsulated mRNA. Fluorescence signals from both conditions are compared to a standard curve of known mRNA concentrations to calculate the mRNA encapsulation efficiency.

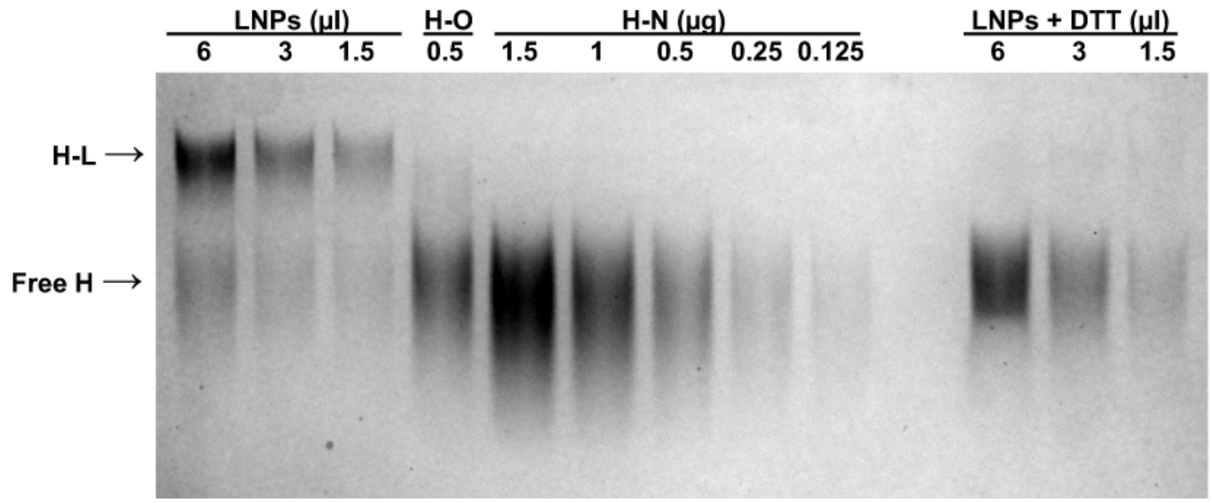

Figure S2: Quantification of surface-conjugated HEP on HEP-LNPs. 13-kDa HEP-LNPs and their parent polymer, HEP-NH_2 (–NH_2 indicating amine functionalization), were analyzed by 8% polyacrylamide gel electrophoresis (PAGE) and stained with Alcian Blue to detect glycosaminoglycans. H-O denotes HEP-OPSS. H-N denotes HEP-NH_2, which was used as the quantification standard due to its more accurate carbohydrate content measurement compared with HEP-OPSS. H-L denotes HEP-LNP. Free H indicates unconjugated (free) HEP. Dithiothreitol (DTT) was used as a reducing agent to cleave the disulfide bond and release HEP from the thiol-lipid component of the LNPs.

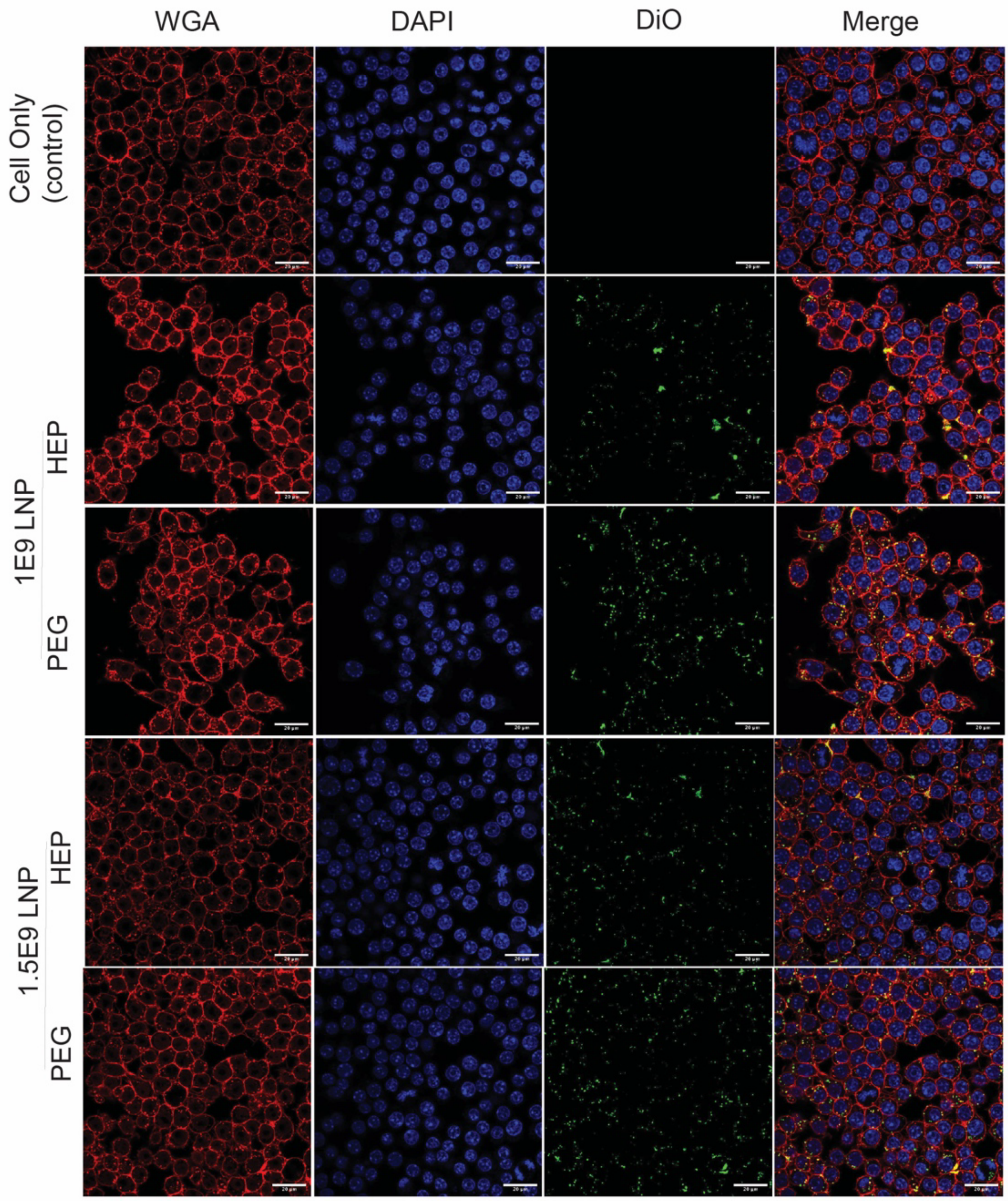

Figure S3: Confocal laser scanning microscopy (CLSM) demonstrating cell uptake of fluorescently labeled mRNA-LNP in RAW 264.7 macrophages. RAW 264.7 murine macrophages were incubated for 24 h at 37°C with HEP- or PEG-coated luciferase mRNA-LNPs with two different amounts of LNPs, i.e. (i) 1.0×10^9 LNPs, or (ii) 1.5×10^9 LNPs. The LNPs numbers were determined with FFF-MALS. The mRNA-LNPs were stained with the lipophilic fluorescent dye DiO. After incubation, the cells were washed, fixed, and stained before being imaged using CLSM. The cell nuclei were visualized with the DNA stain 4′,6 diamidino-2-phenylindole (DAPI, blue), and cell membranes were labeled with wheat germ agglutinin conjugated to CF633 (WGA-CF633, red). The green channel represents the lipophilic dye Vybrant™ DiO used to fluorescently stain the mRNA-LNPs. The scale bars represent 20 µm.

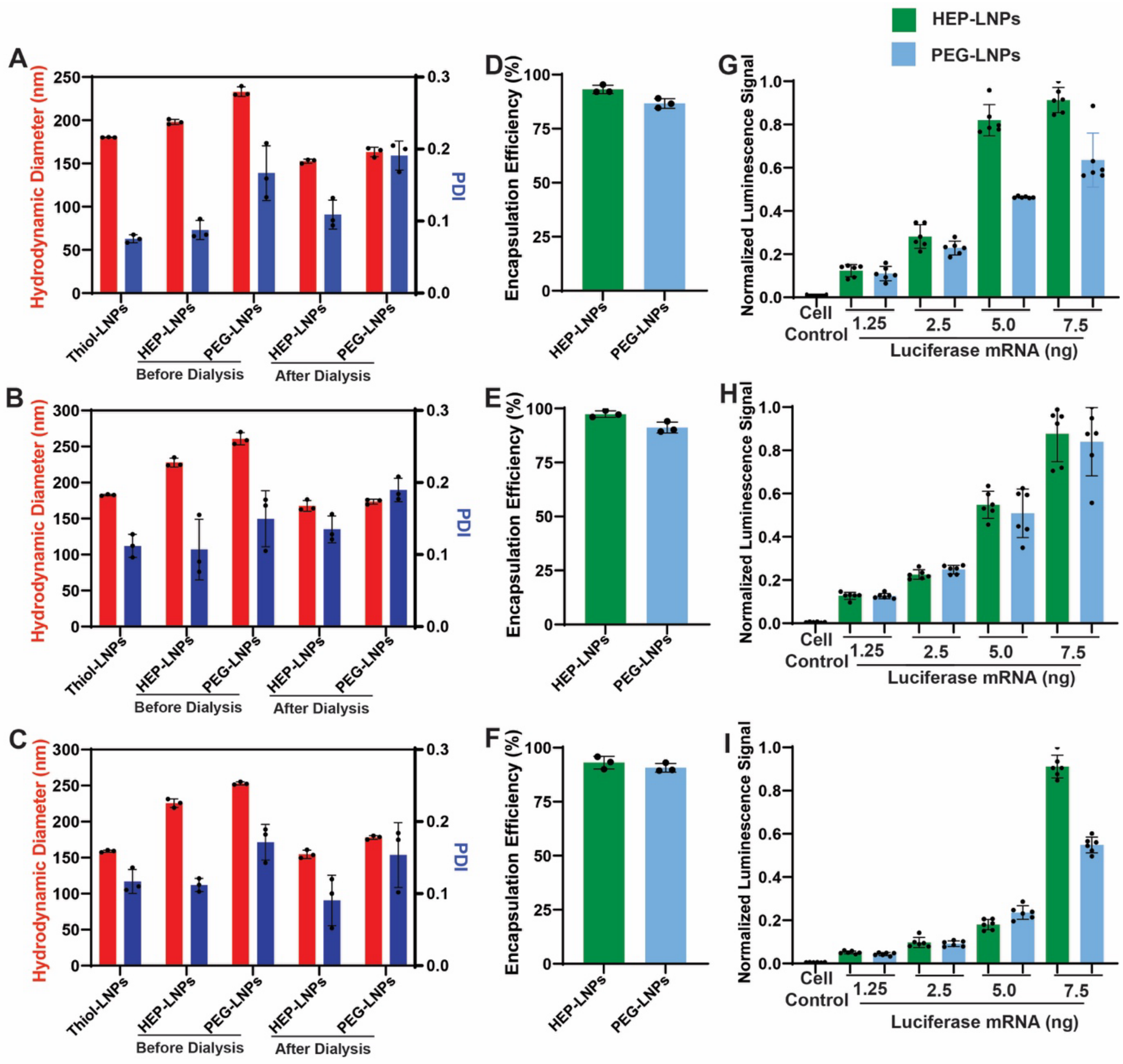

Figure S4: Reproducibility experiments to study the characterization and cell-level transfection of HEP- and PEG-modified firefly luciferase mRNA-LNPs with N/P ratio of 100. (A–C) HDD and PDI of three individual mRNA-LNPs formulations, measured by DLS. Data are presented as mean±SD (n=3). (D–F) %EE of the corresponding mRNA-LNPs as determined with RiboGreen analysis for mRNA. Data are presented as mean±SD (n=3). (G–I) *In vitro* transfection of RAW264.7 murine macrophages with the corresponding mRNA-LNPs. The luciferase-based bioluminescence was measured after 24 hours of incubation. Data are presented as mean±SD (n=6).

Table S1: Concentration of HEP- or PEG-modified mRNA-LNPs (N/P 100) quantified by field-flow fractionation multi-angle light scattering (FFF-MALS).

LNP-Formulation	Replicate Number	LNPs Concentration (LNPs/mL)
HEP-mRNA-LNPs	1	1.2 E11
HEP-mRNA-LNPs	2	1.4 E11
HEP-mRNA-LNPs	3	8.5 E10
PEG-mRNA-LNPs	1	9.9 E10
PEG-mRNA-LNPs	2	1.8 E11
PEG-mRNA-LNPs	3	8.5 E10

Table S2: Concentration of HEP- or PEG-modified mRNA-LNPs (N/P 10) quantified by FFF-MALS.

LNP-Formulation	LNPs Concentration (LNPs/mL)
HEP-mRNA-LNPs	6.4 E10
PEG-mRNA-LNPs	5.9 E10

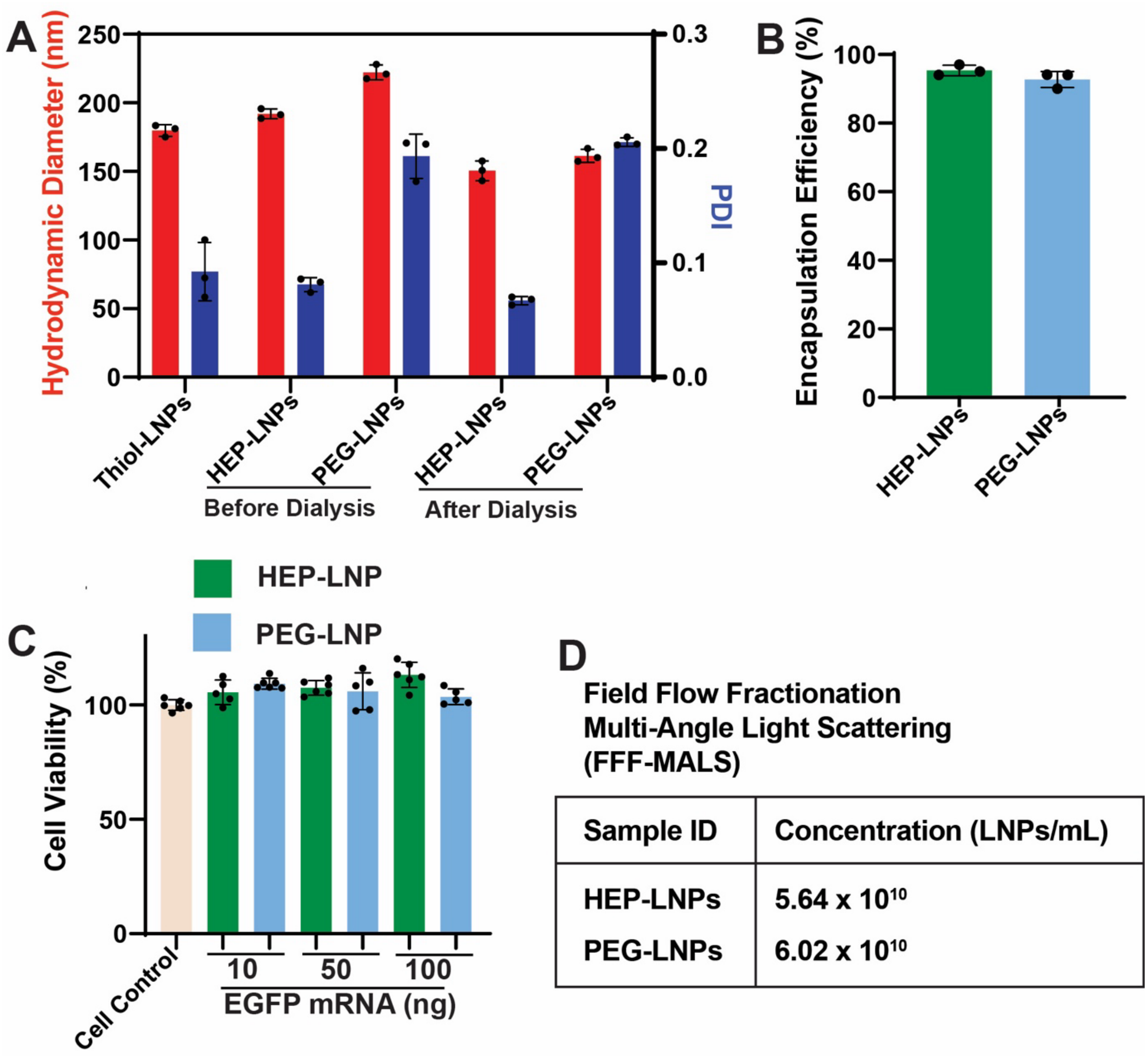

Figure S5: Characterization and *in vitro* cell viability assessment of HEP- and PEG-modified EGFP mRNA-LNPs with N/P ratio of 10. (A) HDD and PDI of HEP- and PEG-modified mRNA-LNPs, measured by DLS. Data are presented as mean±SD (n=3). (B) The %EE results of the mRNA-LNPs. Data are presented as mean±SD (n=3). (C). RAW 264.7 cells were dosed with mRNA-LNPs at 10 ng, 50 ng, and 100 ng of mRNA. Cell viability was quantified using the XTT assay after 24 hours of incubation. Data represent mean±SD (n=5). One-way ANOVA test was used to compare the differences in cell viability. No significant differences (p>0.05) were observed between the experimental samples. (D) mRNA-LNP concentration detected by FFF-MALS.

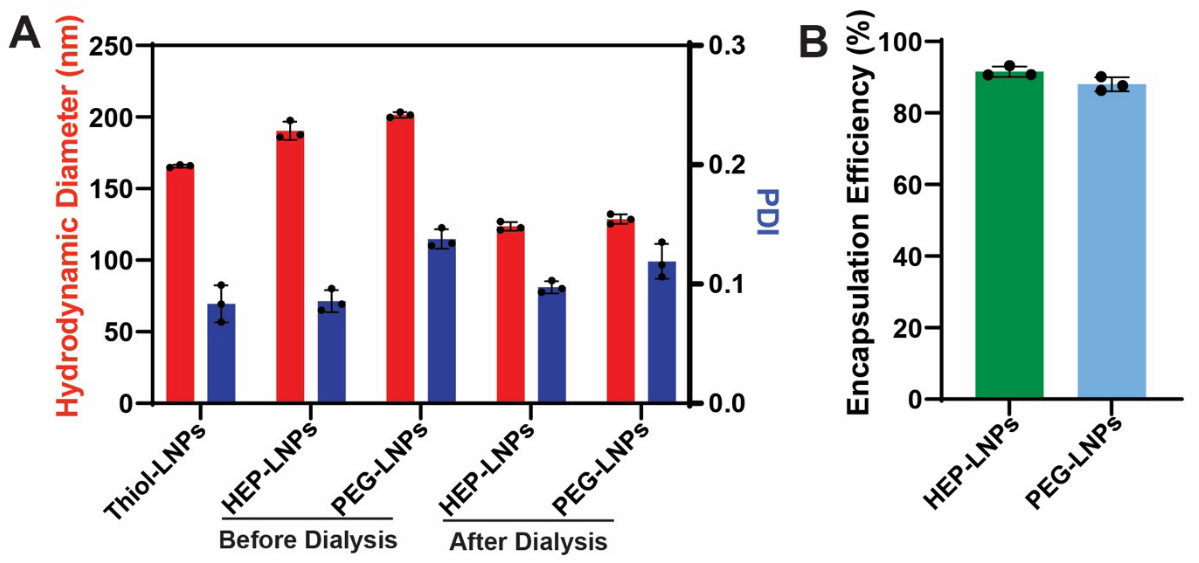

C **Field Flow Fractionation Multi-Angle Light Scattering (FFF-MALS)**

Sample ID	Concentration (LNPs/mL)
HEP-LNPs	5.05×10^{10}
PEG-LNPs	5.82×10^{10}

Figure S6: Characterization of HEP- and PEG-modified Luciferase mRNA-LNPs with N/P ratio of 5. (A) HDD and PDI measurement results of mRNA-LNP with different surface modifications. The data are based on dynamic light scattering measurements. Data are presented as mean±SD (n=3). (B) The %EE results of the mRNA-LNPs. Data are presented as mean±SD (n=3). (C) mRNA-LNP concentration detected by FFF-MALS.

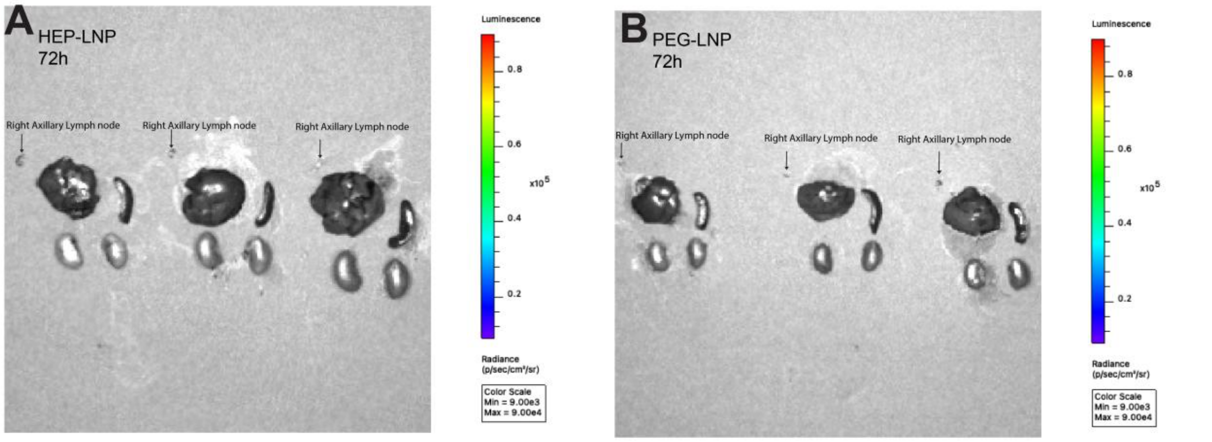

Figure S7: ***Ex vivo*** **bioluminescence imaging of organs from C57BL/6 mice 72 hours after treatment with HEP- or PEG-coated luciferase mRNA-LNPs.** C57BL/6 mice (n=3 per group) were subcutaneously injected with 2 µg of luciferase mRNA-LNPs coated with either HEP or PEG. At 72 hours post-injection, following *in vivo* imaging, mice were euthanized, and major organs, including liver, spleen, kidneys, and axillary lymph nodes, were harvested for *ex vivo* bioluminescence imaging using the IVIS SpectrumCT system. (A) Representative organ bioluminescence from a mouse treated with HEP-mRNA-LNPs. (B) Representative organ bioluminescence from a mouse treated with PEG-mRNA-LNPs. Images were acquired and analyzed with Living Image software. No signal was evident for either LNP formulation.

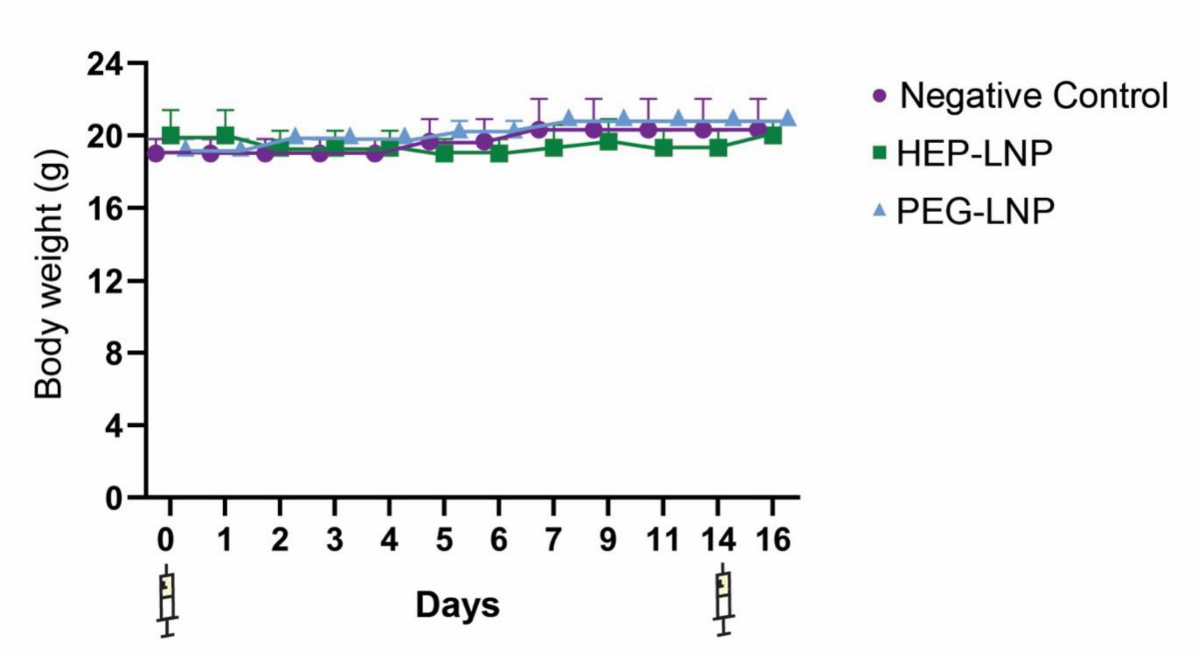

Figure S8: Body weight monitoring of mice following repeated administration of firefly luciferase mRNA-LNPs. C57BL/6 mice were subcutaneously injected with 2 µg of luciferase mRNA-LNPs per mouse, administered twice at a 14-day interval, as indicated by the syringe symbols. The corresponding body weights were recorded over a 16-day period to assess any treatment-related changes.

Table S3: Summary of Histopathological Findings.

	PBS Group	HEP-LNP Group	PEG-LNP Group
Luciferase mRNA Dosage (μg/head)	0	4	4
Tissue and observed content	Incidence (a/b)*		
**Liver**			
No abnormalities detected	3/3	3/3	3/3
**Spleen**			
No abnormalities detected	3/3	3/3	3/3
**Bone Marrow/ Tibia**			
No abnormalities detected	3/3	3/3	3/3
**Kidney**			
No abnormalities detected	3/3	3/3	3/3
**Lung**			
Interstitial widening involving 40% of the tissue; cellular infiltrates; Mild vascular congestion.	1/3	2/3	3/3
Interstitial widening involving 70% of the tissue; cellular infiltrates; Mild vascular congestion.	2/3	1/3	0/3
**Injection site**			
No abnormalities detected	1/3	2/3	0/3
Scattered acute inflammatory cells	2/3	0/3	2/3
Focal fat necrosis	0/3	1/3	0/3
Acute inflammatory cell infiltration surrounding necrotic tissue	0/3	0/3	1/3
**Draining Lymph Node (Left Axillary Lymph Node)**			
No abnormalities detected	2/3	2/3	1/3
Unidentified lymph node	1/3	1/3	2/3

Group summary of histopathological findings (two days post-second injection). Histopathological differences induced by HEP- or PEG-coated luciferase mRNA-LNPs were compared with PBS negative controls in major organs, including the liver, spleen, tibial bone marrow, kidney, lung, injection site, and left axillary draining lymph node. Tissues were examined by hematoxylin and eosin (H&E) staining, and observed changes were recorded as incidences.

*Incidence is expressed as (a/b), where a indicates the number of affected samples and b represents the total number of replicates.

These findings were obtained following two subcutaneous injections of 2 µg of firefly luciferase mRNA-LNPs (100 µL of 1× PBS each), coated with either HEP or PEG, or PBS alone as the negative control, administered 2 weeks apart.
